# A new baby oviraptorid dinosaur (Dinosauria: Theropoda) from the Upper Cretaceous Nemegt Formation of Mongolia

**DOI:** 10.1371/journal.pone.0210867

**Published:** 2019-02-06

**Authors:** Sungjin Lee, Yuong-Nam Lee, Anusuya Chinsamy, Junchang Lü, Rinchen Barsbold, Khishigjav Tsogtbaatar

**Affiliations:** 1 School of Earth and Environmental Sciences, Seoul National University, Seoul, South Korea; 2 Department of Biological Sciences, University of Cape Town, Cape Town, South Africa; 3 Institute of Geology, Chinese Academy of Geological Sciences, Beijing, China; 4 Institute of Paleontology and Geology, Mongolian Academy of Sciences, Ulaanbaatar, Mongolia; Universidade Federal da Bahia, BRAZIL

## Abstract

Recent discoveries of new oviraptorosaurs revealed their high diversity from the Cretaceous Period in Asia and North America. Particularly, at the family level, oviraptorids are among the most diverse theropod dinosaurs in the Late Cretaceous of Mongolia and China. A new oviraptorid dinosaur *Gobiraptor minutus* gen. et sp. nov. from the Upper Cretaceous Nemegt Formation is described here based on a single holotype specimen that includes incomplete cranial and postcranial elements. The most prominent characters of *Gobiraptor* are its thickened rostrodorsal end of the mandibular symphysis and a rudimentary lingual shelf on each side of the dentary. Each lingual shelf is lined with small occlusal foramina and demarcated by a weakly developed lingual ridge. This mandibular morphology of *Gobiraptor* is unique among oviraptorids and likely to be linked to a specialized diet that probably included hard materials, such as seeds or bivalves. The osteohistology of the femur of the holotype specimen indicates that the individual was fairly young at the time of its death. Phylogenetic analysis recovers *Gobiraptor* as a derived oviraptorid close to three taxa from the Ganzhou region in southern China, but rather distantly related to other Nemegt oviraptorids which, as the results of recent studies, are also not closely related to each other. *Gobiraptor* increases diversity of oviraptorids in the Nemegt Formation and its presence confirms the successful adaptation of oviraptorids to a mesic environment.

## Introduction

Oviraptorosauria is an unusual group of maniraptoran theropods with distinctive anatomical characters such as a deep and short skull, edentulous jaws in derived forms, a short tail, and pneumatized proximal caudal vertebrae [1–4]. The origin of oviraptorosaurs is generally assumed to be from Asia based on their earliest records from the Lower Cretaceous Yixian Formation of China [5–7]. Derived forms mostly appeared in the Late Cretaceous [4, 8] when they dispersed throughout Asia and North America [9, 10]. Within the clade Oviraptorosauria, three derived families have been recognized: Avimimidae [11], Caenagnathidae [12], and Oviraptoridae [1]. Avimimids are comprised of a single genus that includes two species from the Nemegt Formation of Mongolia [10, 11, 13] while caenagnathids and oviraptorids show high level of diversity that has especially been bolstered by recent discoveries from the Nanxiong Formation of the Ganzhou region in southern China [14–20]. Interestingly, oviraptorids are restricted to Asia although they are more diverse than caenagnathids which are reported from both Asia and North America [8, 10]. However, most of the caenagnathids are represented by fragmentary materials [12, 21–34] with only a few exceptions [8, 9, 35, 36] compared with the numerous nearly complete skeletons of oviraptorids [4, 19, 20, 37–40].

**Fig 1:**
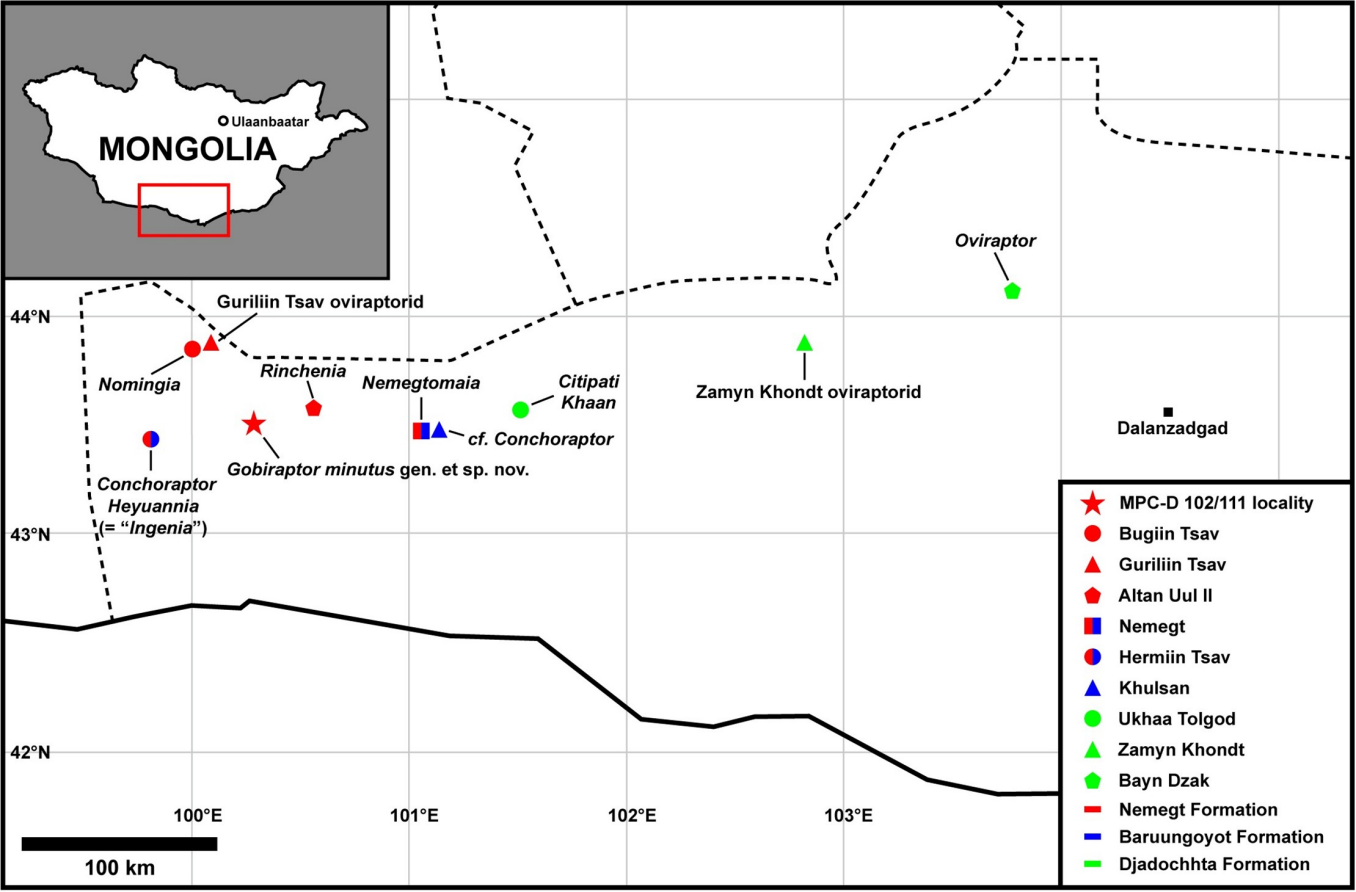
Map showing the occurrences of oviraptorids in the southern Gobi Desert of Mongolia. The map was generated using Simplemappr (www.simplemappr.net) before modified.

Although the Nanxiong Formation is the most productive formation with regard to the number of oviraptorid taxa [20], the Gobi Desert of Mongolia, including the classic Nemegt locality (Fig 1), has also yielded abundant oviraptorids [1, 2, 4, 37, 40–49]. Despite this high diversity, oviraptorid occurrences have been relatively rare in the Altan Uul area [10, 50]. In 2008, an oviraptorid specimen was found along with other theropod skeletons during the Korea-Mongolia International Dinosaur Expedition (KID) from the Nemegt Formation of Altan Uul III, Mongolia (Fig 1, S1 Fig). The specimen is described here as a new oviraptorid taxon *Gobiraptor minutus* gen. et sp. nov., which is mainly characterized by its peculiar mandibular morphology. *Gobiraptor minutus* increases the diversity of oviraptorids in the Nemegt Formation and together with the unnamed Guriliin Tsav oviraptorid [10] that may represent a new taxon, shows that oviraptorids were exceptionally diverse in the Gobi Desert with at least 10 taxa. Additionally, the discovery of *Gobiraptor minutus* provides valuable insight into the evolution and dietary adaptations of the Nemegt oviraptorids and their abundance in a mesic environment.

## Methods

### Repository of the holotype specimen

The holotype specimen (MPC-D 102/111) is permanently held in the Institute of Paleontology and Geology in Ulaanbaatar, Mongolia.

### Phylogenetic analysis

A phylogenetic analysis was performed to obtain the position of *Gobiraptor minutus* within the clade Oviraptorosauria. The character list and data matrix (S1 Text) used in this study were modified from that of Lü et al. [20]. The modifications include the following: unordering five ordered characters (82, 89, 183, 196, and 207) as suggested by Funston and Currie [8]; correcting an error in the data matrix of *Yulong mini* (character state 102:2 to 102:1); changing the name of *Ingenia yanshini* to *Heyuannia yanshini* following Funston et al. [10]; changing the character states of *Gigantoraptor erlianensis* (195:0 to 195:1) and *Heyuannia yanshini* (94:1 to 94:0) based on the anatomical descriptions of each of these two species in Ma et al. [51] and Funston et al. [10], respectively; combining *Caenagnathus sternbergi*, *Macrophalangia canadensis*, and *Chirostenotes pergracilis* as well as Alberta dentary morph 3 and *Leptorhynchos elegans* replacing *Elmisaurus elegans* following Funston and Currie [8]; removal of *Ojoraptorsaurus boerei* also following Funston and Currie [8]; adapting the updated data matrices of *Caenagnathus collinsi*, *Caenagnathasia martinsoni*, *Elmisaurus rarus*, and *Leptorhynchos elegans* in Funston and Currie [8]; incorporation of *Gobiraptor minutus* to the data matrix. Including *Gobiraptor minutus*, 42 taxa with 257 characters were analyzed in TNT version 1.5 [52]. An identical traditional search with the one in Lü et al. [20] (Wagner trees; swapping algorithm: tree bisection-reconnection; random seeds: 1,000; replicates: 1,000; trees to save per replication: 10) was run, and 24 most parsimonious trees (MPTs) with 652 steps were produced (consistency index [CI]: 0.448, retention index [RI]: 0.647). The ‘Bremer.run’ script was used in TNT [52] to calculate the Bremer support values on each node of the strict consensus tree of the 24 MPTs. The tree data were then transferred to Winclada version 1.00.08 [53] to generate the tree image.

### Osteohistological examination

A piece from the mid-shaft of the right femur was sampled and embedded in a polyester resin. Two histological thin sections (30 microns and 25 microns) were prepared following standard petrographic techniques [54]. The thin sections were studied under a Nikon E200 and a Zeis AXIO petrographic microscope, Photomicrographs were taken with a Nikon camera using NIS elements (version 4). Terminology used for the histological descriptions are *sensu* Chinsamy-Turan [54].

### Nomenclatural acts

The electronic edition of this article conforms to the requirements of the amended International Code of Zoological Nomenclature, and hence the new names contained herein are available under that Code from the electronic edition of this article. This published work and the nomenclatural acts it contains have been registered in ZooBank, the online registration system for the ICZN. The ZooBank LSIDs (Life Science Identifiers) can be resolved and the associated information viewed through any standard web browser by appending the LSID to the prefix "http://zoobank.org/". The LSID for this publication is: urn:lsid:zoobank.org:pub:F3B7BF15-2CD5-4FD6-983C-809B56FB0B59. The electronic edition of this work was published in a journal with an ISSN, and has been archived and is available from the following digital repositories: PubMed Central, LOCKSS.

### Institutional abbreviation

MPC, Institute of Paleontology and Geology, Mongolian Academy of Sciences, Ulaanbaatar, Mongolia.

## Results

### Systematic paleontology

Dinosauria Owen, 1842 [55]

Theropoda Marsh, 1881 [56]

Maniraptora Gauthier, 1986 [57]

Oviraptorosauria Barsbold, 1976 [1]

Oviraptoridae Barsbold, 1976 [1]

*Gobiraptor minutus* gen. et sp. nov.

LSID for the genus: urn:lsid:zoobank.org:act:116FF31F-8492-4BB4-9961-53E586A136EC

LSID for the species: urn:lsid:zoobank.org:act:53F0E7D7-EB76-4B8F-8801-AED4FE792E8C

#### Etymology

The generic name *Gobiraptor* is a combination of ‘Gobi’ which refers to the Gobi Desert where the holotype specimen was found and ‘raptor’ which is Latin for thief. The specific name ‘*minutus’* is Latin for small and refers to the small size of the holotype specimen.

#### Holotype

**Fig 2:**
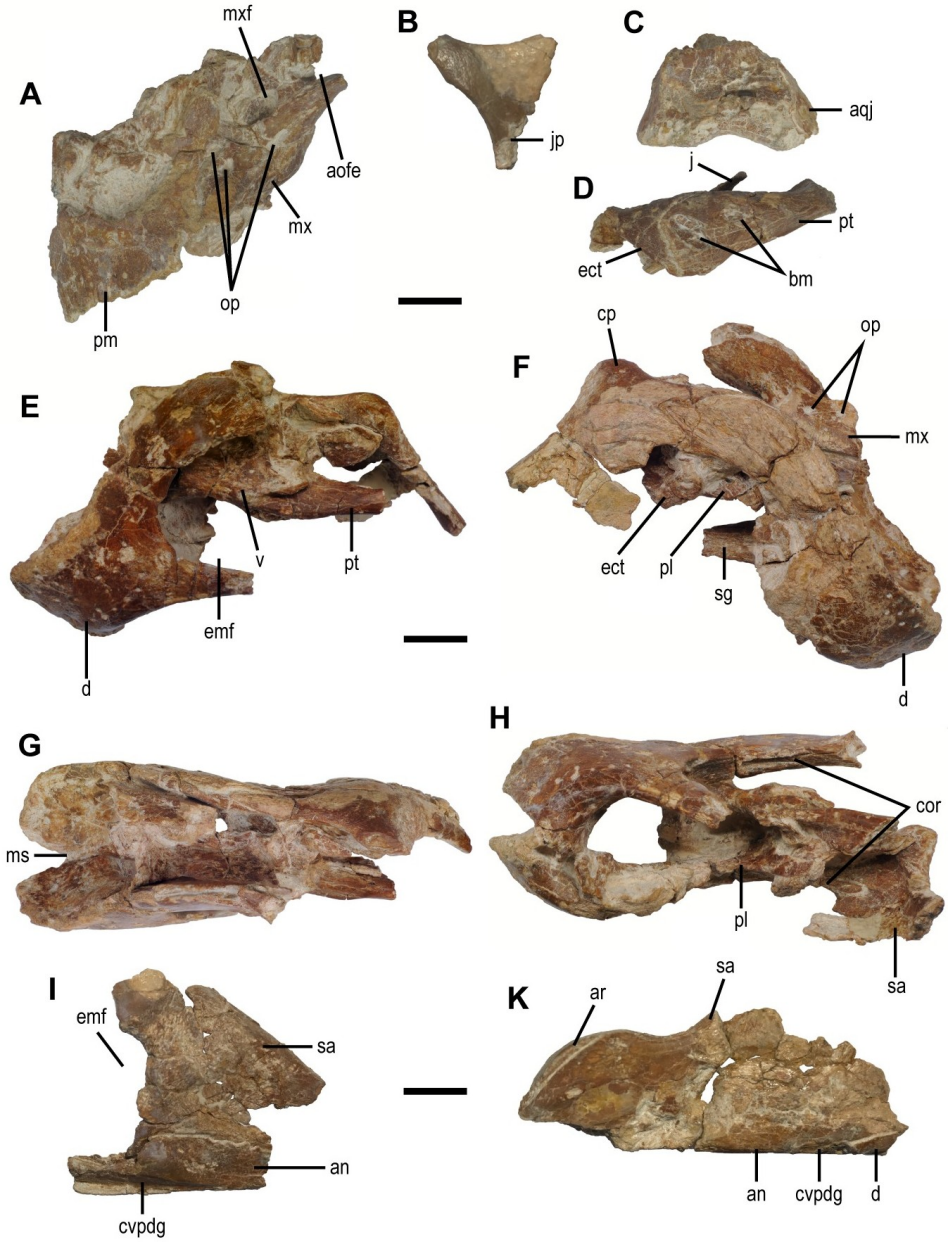
Cranial elements of the holotype specimen (MPC-D 102/111) of *Gobiraptor minutus* gen. et sp. nov. (A) Left premaxilla and maxilla in lateral view. (B) Left postorbital in lateral view. (C) Right quadrate in caudal view. (D) Left ectopterygoid and pterygoid in lateral view. (E-H) Rostral region of the mandible in left lateral (E), right lateral (F), dorsal (G), and oblique ventral (H) views. (I) Left surangular and angular in lateral view. (K) Caudal region of the right mandibular ramus in lateral view. Abbreviations: an, angular; aofe, antorbital fenestra; aqj, articular surface for quadratojugal; ar, articular; bm, bite mark(s); cor, coronoid bone; cp, coronoid process; cvpdg, groove for the caudoventral process of dentary; d, dentary; ect, ectopterygoid; emf, external mandibular fenestra; j, jugal; jp, jugal process of postorbital;ms, intermandibular suture; mx, maxilla; mxf, maxillary fenestra; pl, palatine; pm, premaxilla; pt, pterygoid; sa, surangular; sg, groove for splenial; v, vomer. Scale bars equal 1 cm.

**Fig 3:**
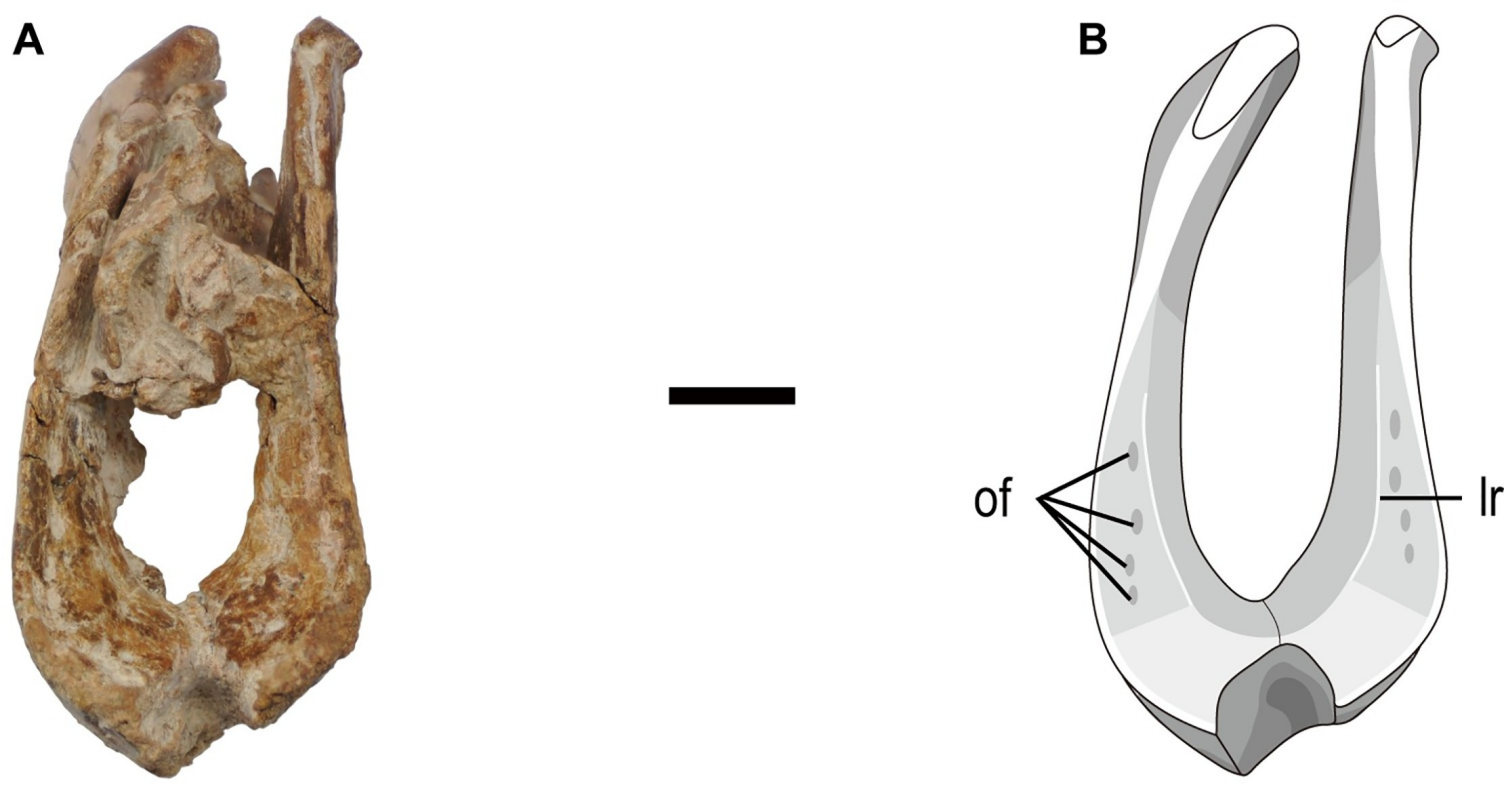
Rostral part of the mandible of the holotype specimen (MPC-D 102/111) of *Gobiraptor minutus* gen. et sp. nov. (A) Mandible in rostral view. (B) Interpretive drawing of A. The cranial elements caught between the mandibular rami are omitted. Abbreviations: lr, lingual ridge; of, occlusal foramen. Scale bar equals 1 cm.

**Fig 4:**
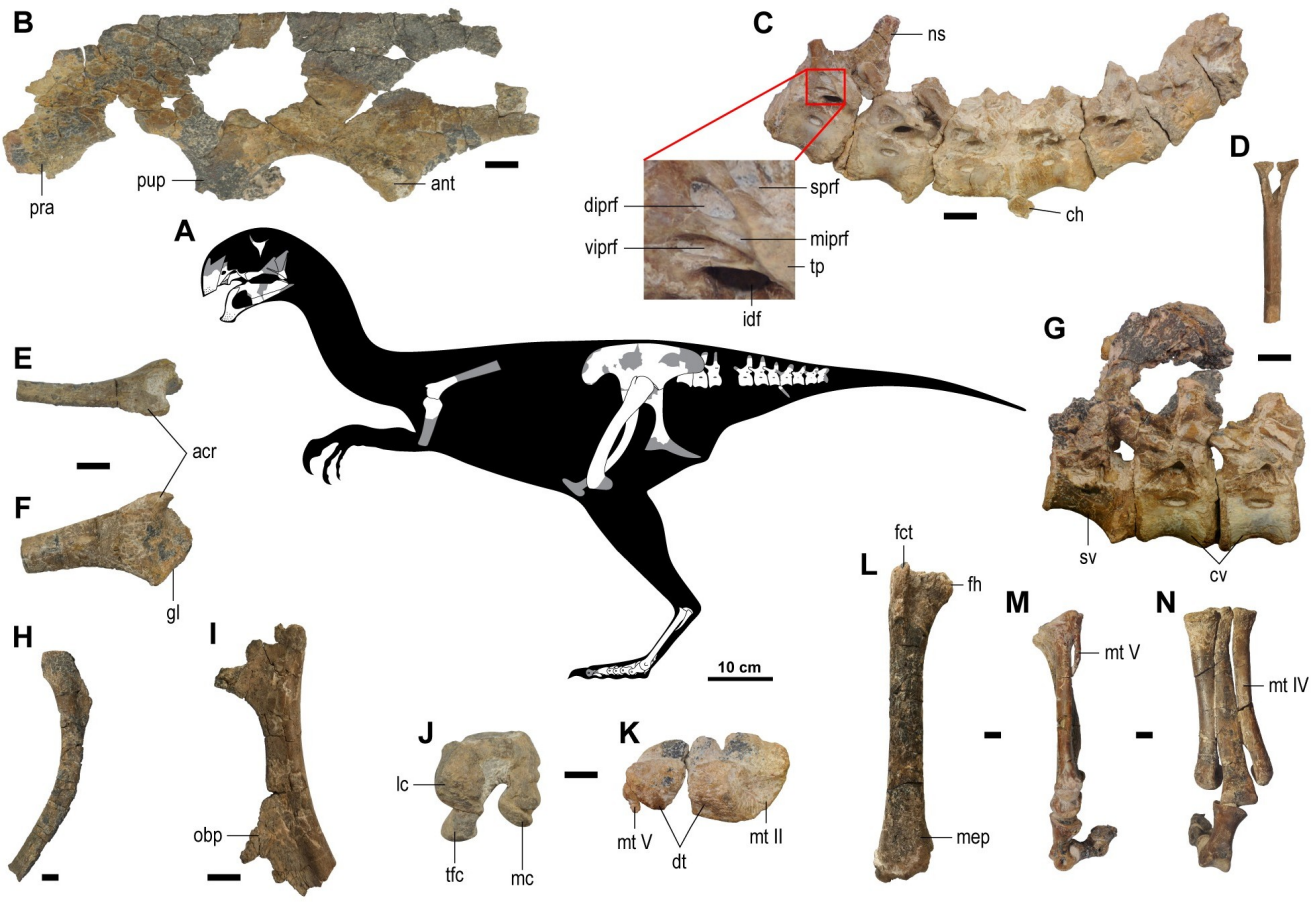
Postcranial elements of the holotype specimen (MPC-D 102/111) of *Gobiraptor minutus* gen. et sp. nov. (A) Skeletal reconstruction in left lateral view (missing and damaged portions of the bones in gray). (B) Left ilium in lateral view. (C) Proximal caudal vertebrae in left lateral view with close-up of the infraprezygapophyses. (D) Chevron in cranial view. (E-F) Right scapula in dorsal (E) and lateral (F) views. (G) Last sacral and the two proximalmost caudals in left lateral view. (H) Right pubis in medial view. (I) Right ischium in lateral view. (J) Right femur in distal view. (K) Left metatarsus and distal tarsals in proximal view. (L) Right femur in cranial view. (M-N) Left metatarsus in lateral (M) and dorsal (N) views. Abbreviations: acr, acromion process; ant, antitrochanter; ch, chevron; cv, caudal vertebra(e); diprf, dorsal infraprezygapophyseal fossa; dt, distal tarsal(s); fct, cranial trochanter of femur; fh, femoral head; gl, glenoid fossa; idf, infradiapophyseal fossa; lc, lateral condyle; mc, medial condyle; mep, medial epicondyle; miprf, middle infraprezygapophyseal fossa; mt II, metatarsal II; mt IV, metatarsal IV; mt V, metatarsal V; ns, neural spine; obp, obturator process; pra, preacetabular process; pup, pubic peduncle; sprf, supraprezygapophyseal fossa; sv, sacral vertebra; tfc, tibiofibular crest; tp, transverse process; viprf, ventral infraprezygapophyseal fossa. Scale bars equal 10 cm in (A); 1 cm in (B-N).

The holotype specimen (MPC-D 102/111) (Figs 2–4, S2 and S3 Figs) consists of mostly incomplete cranial and postcranial elements including ventral parts of the premaxillae and maxillae, a tip of the right jugal, fused vomer, parts of articulated pterygoids and ectopterygoids, incomplete right palatine, central part of the left postorbital, partial right quadrate and quadratojugal, incomplete lower jaw, with most of its elements broken, the last sacral vertebra which is articulated with the two proximalmost caudal vertebrae, articulated but incomplete proximal caudal vertebrae, fragments of chevrons, partial right scapula and humerus, incomplete pelvic girdles, nearly complete both femora, complete left metatarsus with distal tarsals 3 and 4, incomplete left pedal digits I, III, and IV, and several unidentified fragments. MPC-D 102/111 was also found with other theropod skeletons including postcranial elements of alvarezsaurids and larger oviraptorids.

#### Type locality and horizon

Altan Uul III [58–62], Ömnögovi Province, Mongolia (Fig 1, S1 Fig). Upper Cretaceous Nemegt Formation [58–60, 62–64].

#### Diagnosis

*Gobiraptor minutus* is an oviraptorid dinosaur diagnosed by the following unique set of characteristics (autapomorphies are marked with an asterisk): a flat articular surface for the quadratojugal on the quadrate*; rostrocaudally elongate dentary rostral to the external mandibular fenestra; extremely thickened rostrodorsal end of the mandibular symphysis with a caudal expansion of its dorsal surface *; a rudimentary lingual triturating shelf on each dentary bearing small occlusal foramina*; a weakly developed lingual ridge on each lingual shelf*; absence of any prominent symphyseal ventral process of the dentary; coronoid bone present; the rostral end of the coronoid bone wedging into the ventral surface of the dorsal ramus of the dentary*; cranial trochanter of the femur separated from the greater trochanter with a distinct furrow between them.

*Gobiraptor minutus* differs from *Nemegtomaia barsboldi* [10, 47, 65] in that there is a non-mobile joint between the quadrate and quadratojugal, the rostral end of the mandibular symphysis is much thicker, there are weakly developed lingual shelves and ridges, the dentary shows no deflection at the rostral margin of the external mandibular fenestra, and a distinct ventral process on the mandibular symphysis is not present.

*Gobiraptor minutus* is different from *Heyuannia* (“*Ingenia*”) *yanshini* [10, 42] in that the last sacral vertebra bears a pleurocoel on each lateral surface of the centrum, the scapula has a ventrally directed glenoid, the finger-like cranial trochanter of the femur is well developed and separated from the greater trochanter, and the distal shaft of the metatarsal IV is laterally deflected.

*Gobiraptor minutus* is differentiated from *Conchoraptor gracilis* [10, 44] by the maxilla being more steeply inclined, the quadrate lacking the lateral cotyle for the quadratojugal, the vomer with a caudal process between the two pterygoids, and no fusion between the palatine and pterygoid.

*Gobiraptor minutus* primarily differs from *Rinchenia mongoliensis* (= *Oviraptor mongoliensis*) [4, 10, 44, 66] in that the premaxilla has a relatively elongate tomial margin, the rostrodorsal end of the mandible is much thickened, the mandibular symphysis does not have a prominent ventral process, the ilium has a straight dorsal margin, the cranioventral margin of the preacetabular process is rounded, and the cranial trochanter of the femur is not fused with the greater trochanter.

*Gobiraptor minutus* is distinguished from *Citipati osmolskae* [37, 67] mainly by the well-developed caudal process of the quadratojugal, the dentary with the rostrocaudally elongate symphyseal region, the coronoid bone whose rostral end is ventrally placed to the caudodorsal ramus of the dentary, and the lateral surface of the articular that is not completely covered by the surangular.

*Gobiraptor minutus* also differs from *Nomingia gobiensis* [2, 10] in that the preacetabular process of the ilium does not have a convex dorsal margin, the cuppedicus fossa is not visible in lateral view, the pubic shaft is more concave cranially, and there is no fusion between the cranial trochanter and the greater trochanter on the femur.

### Description

#### Skull and mandible

The skull and mandible (Figs 2 and 3, S2 Fig, see S1 Table for measurements) of the holotype specimen of *Gobiraptor* are incompletely preserved and most of the preserved cranial elements are distorted or crushed by compression from the lateral side. A fragment of the right maxilla, the vomer, and the right palate bones are caught in between the mandibular branches detached from the skull. The cranial elements generally show a clear suture at each border between individual bones. Except for the partially preserved postorbital, the upper and middle regions of the skull are missing in the holotype.

The premaxilla (Fig 2A, S2A and S2B Fig) is rostrocaudally elongate in lateral view and the rostral margin is vertical as in *Citipati osmolskae* [67] or *Conchoraptor* [4, 10]. Whether the premaxillae are fused with each other is not certain since their rostral end is distorted. This distortion made a triangular gap at the tip of the rostrum. The premaxilla is also edentulous and has an oblique tomial margin whose crenulation is obscured by weathering. The upper region of both premaxillae is missing, thus the exact location of the external nares or the location of the border between the premaxilla and nasal cannot be inferred. There are irregularly placed small nutrient foramina on the lateral surface of the premaxilla above the tomial margin. The maxillary process of the premaxilla caudodorsally extends to probably meet the nasal at the dorsal end. The palatal surface of the premaxilla is concave and U-shaped in ventral view. Along the tomial margin, there is a row of small foramina which would have met the occlusal grooves on the dentary when the beak was closed (S2A Fig).

The rostrocaudally short maxilla (Fig 2A, S2A Fig) is edentulous as the premaxilla. The right maxilla is fragmentary and the more complete left one has a crushed lateral surface. The lateral surface of the maxilla contacts the premaxilla rostrally but it is broken along the border. The antorbital fossa is not recognizable because of the crushed surface. The maxilla does not form a continuous ventral margin with the tomial margin of the premaxilla but ascends in a greater angle in lateral view. A maxillary fenestra is present on the lateral surface rostral to the antorbital fenestra but its rim is lost except for the caudoventral margin. The caudal border of the maxillary fenestra is comprised of the interfenestral bar which also constitutes the rostral margin of the antorbital fenestra. Caudal to the interfenestral bar, the maxilla extends caudally as a narrow splint to make up the ventral margin of the antorbital fenestra. There are two openings on the lateral surface of the right maxilla (Fig 2F) and three on the left (Fig 2A). The openings on the right maxilla are circular and smaller than the ones on the left. Between the two openings on the right maxilla, the caudal one is larger than the rostral one. The openings on the left maxilla are similar in size and shape being subtriangular. Two of them are very close to each other and located near the rostral margin of the maxilla while the third one is right beneath the maxillary fenestra. Some of these could be accessory openings which have also known in other oviraptorids. On the palatal surface of each maxilla is a rostrocaudally elongate ridge that must have bordered the premaxilla rostrally.

The partially preserved postorbital (Fig 2B) is a triradiate bone but none of the three processes is complete. The frontal process of the postorbital is broken at its base although it shows a rostrodorsal orientation, which is typical of oviraptorids. The mediolaterally thin squamosal process extends caudodorsally but its tip is missing. The rostral margin of the postorbital forms the caudodorsal orbital rim. It is caudally concave and caudomedially slanted. The jugal process of the postorbital is rostrocaudally narrow but mediolaterally long having a subrectangular cross section.

The quadratojugal (S2C Fig) tightly adheres to the quadrate without a distinct suture. The dorsal and rostral processes of the quadratojugal are perpendicular to each other. They are broken off near the quadratojugal body while the caudal process is well preserved and extends caudoventrally beyond the quadrate. The medial articular surface of the quadratojugal for the quadrate is distinctly concave.

The quadrate (Fig 2C, S2C Fig) is missing its dorsal part of the shaft. The quadrate becomes narrower dorsally while it widens medially. The mandibular articular surface of the quadrate is saddle-shaped and divided into two distinct condyles by a longitudinal groove at the center. The lateral condyle extends slightly further ventrally than the medial condyle. Dorsolateral to the lateral condyle is the articular surface for the quadratojugal. It is flat lacking a concavity described by Maryanska and Osmólska [68] or a convex surface in *Nemegtomaia* [47]. The caudal surface of the quadrate is prominently concave and the broad medial surface is nearly flattened.

The palate (Fig 2D–2H) of *Gobiraptor* generally shows a typical oviraptorid morphology described by Elzanowski [69] and Osmólska et al. 2004 [4]. The fused vomer (Fig 2E) forms a round ventral process at its rostral end but the rostral tip is obscured by the dentary and matrix. The vomer has a dorsal expansion which meets the maxilla although the border is worn off. Each lateral surface of the ventral process of the vomer is concave and steeply slanted bordering a round ridge caudodorsally. Caudal to these ridges, the vomer has a medially concave lateral surface which gradually expands dorsoventrally in lateral view. The ventral surface of the vomer becomes flat caudally. At its caudal end, the vomer is tightly wedged by the pterygoids and separates them with a short caudal process. The vomer also contacts the palatine laterally together forming the choana. The preserved specimen only shows the right choana whose rostral border is not visible because of matrix.

The left palatine is entirely missing while the right palatine (Fig 2F and 2H) is preserved articulating with the pterygoid and the ectopterygoid although it is heavily eroded. The exposed part of the palatine is a thin lateral ramus which is visible in lateral and ventral views. The lateral ramus extends to probably meet the maxilla and dorsomedially to meet the vomer. The contact between the palatine and the maxilla, however, is obscured by the dentary and matrix. The palatine also contacts the pterygoid and the ectopterygoid caudodorsally but it is not fused to them showing a clear suture at the border. The suborbital fenestra that is present in *Citipati* [67] and *Conchoraptor* [69] is not visible.

The pterygoid (Fig 2D, 2E and 2H) shows a typical morphology of oviraptorids. The palatal ramus of the pterygoid has concave dorsal and ventral surfaces, the latter being deeper. The pterygoid contacts the ectopterygoid rostrally and dorsally forming a rostrocaudally elongate pterygoid-ectopterygoid bar. The suture between the pterygoid and the ectopterygoid is distinct and V-shaped in dorsal and lateral views. Rostrally, the pterygoid-ectopterygoid bar has a deep ventral flange which is mainly formed by the pterygoid. Caudal to the flange the pterygoid becomes slender. The rod-like caudal tip of the right jugal is broken off and adheres to the medial surface of the left pterygoid. There are two elliptical bite marks on the lateral surface of the left pterygoid (Fig 2D). Between the two bite marks, the rostral one is much larger and deeper. The bite marks do not show any sign of healing suggesting that either the animal died as a result of predation or it was scavenged after death.

The ectopterygoid (Fig 2D and 2F) extends rostrodorsally to meet the maxilla and jugal although these contacts are not preserved. Caudal to this ascending process, the ectopterygoid dorsoventrally expands and contacts the pterygoid caudally in lateral view. The thin dorsal surface of the ectopterygoid is concave and overlies the palatine and the pterygoid.

The mandible (Figs 2E–2K and 3, S2D–S2F Fig) is severely distorted and broken into several pieces although the rostral region is relatively well preserved. The preserved mandibular elements are mostly incomplete including both dentaries, surangulars, coronoid bones, angulars, splenials, prearticulars, and the right articular. The morphology of the mandible shows oviraptorid features, namely the large external mandibular fenestra and the distinct coronoid processes on each surangular.

The unfused dentary (Figs 2E–2H and 3, S2D Fig) is edentulous, deep and marked by numerous small nutrient foramina on the rostral and lateral surfaces. Like the premaxilla, the two dentaries are compressed in the same direction and as a result, they are distorted and broken having a V-shaped gap at the rostral tip (Figs 2G, 2H and 3). The symphyseal region of the dentary is greatly downturned at an angle of approximately 32° to the ventral ramus when measured as done in Ma et al. [51] and its rostrodorsal tip is only slightly upturned. The mandibular symphysis (Figs 2G and 3) is unique among oviraptorids, its rostrodorsal end being extremely thickened. Its dorsal surface extends caudally with small pits. The dentary also has a weakly developed lingual ridge defining each rudimentary lingual shelf that extends caudally from the symphysis (Fig 3). These ridges do not meet each other at the middle and they are not well developed like those in derived caenagnathids [36]. Each lingual shelf bears at least four elliptical occlusal foramina (Fig 3). The caudal margin of the mandibular symphysis, together with that of the lingual shelves, forms a U-shaped margin. Caudal to this margin, a slightly concave surface that is nearly perpendicular to the dorsal surface of the symphysis slopes down to the ventral margin of the dentary at the middle (S2D Fig). Lateral to this surface is a large fossa near the rostral end of the splenial in case of the left dentary. Caudally, there is also a deep fossa on the ventral surface of the lingual shelf. There are likely to be another pair of fossae on the right dentary but this region is covered by matrix. The large external mandibular fenestra is semicircular with smoothly curved rostrodorsal margin and mostly formed by the dentary and surangular (Fig 2E, 2F and 2I). The dentary strongly thins towards the rostral margin of the external mandibular fenestra not having a deflection known in *Nemegtomaia* [47]. The external mandibular fenestra divides the dentary into two rami. The mediolaterally thicker caudodorsal ramus ascends making a weak S-shape in lateral view until it is bifurcated by the surangular into lateral and medial branches. At their borders, the dentary and surangular are tightly joined together making a zig-zagged suture. The thin and splint-like coronoid bone which has been known only in *Citipati* [67] dorsally wedges into the ventral surface of the caudodorsal ramus of the dentary at its rostral end (Fig 2H). However, this is unlike in *Citipati* where it is on the medial surface of the caudodorsal ramus [67]. The coronoid bone twists as it extends to the medial surface of the surangular along a shallow groove which is below the coronoid process. The mediolaterally thin caudoventral ramus of the dentary is elongate and must have extended caudally beyond the caudal margin of the external mandibular fenestra like *Rinchenia* [10] along the shallow groove on the lateral surface of the angular. On the medial surface of the caudoventral ramus, there is a shallow groove for the splenial.

The thin splenial (S2E Fig) is poorly preserved. Both left and right splenials are missing their rostral parts, but the groove on the medial surface of the left dentary indicates that their rostral ends must have reached below the symphyseal shelf. This groove for the splenial extends along the caudodorsal ramus of the dentary to the medial surface of the angular where the splenial overlies the prearticular. The splenial tapers caudally forming a pointed end at which the prearticular twists so that its broad surface faces medially.

Both surangulars are preserved in MPC-D 102/111 but the left surangular (Fig 2I) is fragmentary. The broken but better preserved right surangular (Fig 2F–2H and 2K, S2C–S2F Fig) is relatively thick along the dorsal margin until it meets the articular although it is broken and missing its middle region. The prominent coronoid process is dorsomedially oriented and ventrally forms a ridge on the medial surface. Below the coronoid process is the convex lateral surface caudal to which is a low ridge in contrast to the concave medial surface. The surangular gently descends caudally from the coronoid process to meet the articular but does not completely cover it in lateral view having a distinct suture along the border unlike *Citipati* [67]. As in other oviraptorids, there is a thin process which protrudes into the external mandibular fenestra but it is broken at its base. Ventral to the dorsal margin, the surangular becomes thin and contacts the angular on the lateral surface and the prearticular on the medial surface ventrally also with clear sutures. The surangular is not fused with the articular and does not contribute to the mandibular articulation surface. In dorsal view (S2F Fig), the suture between the surangular and articular is V-shaped. The lateral surface which incompletely covers the articular is flat but has a minute bump near the caudal end of the surangular-articular suture (Fig 2K). There is no visible adductor fossa or a foramen on the surangular but it could be due to poor preservation.

The preserved angulars (Fig 2I and, 2K, S2E Fig) are fragmentary but much of the morphological information is not lost. The angular is well exposed in lateral view and generally thin but on its lateral surface has a small mound right below the rostral end of the border with the surangular. Ventrally the angular also has a shallow depression on the lateral surface for the caudoventral ramus of the dentary. On the medial surface, a groove for the prearticular lies under the splenial and its associated groove. The ventral surface of the angular is flat, maintaining almost constant mediolateral width until it is invaded by the prearticular.

The rostrocaudally elongate mandibular articulation surface is entirely formed by the articular and its shape is semicircular in dorsal view (S2F Fig). Around the midline of the articulation surface, there is a low longitudinal ridge which must have articulated with the groove between the two condyles of the quadrate. This ridge divides the articulation surface into two glenoids which are dorsally convex and probably allowed propalinal movement at the jaw joint. The medial glenoid is slender and nearly flat in contrast to the massive lateral glenoid which does not laterally extend beyond the level of the lateral surface of the surangular. Below the mandibular articulation, the medial surface of the articular is partially covered by the surangular and prearticular.

The prearticular (S2E Fig) is rod-like rostrally but soon twists before it dorsoventrally expands near the articular to become a major element of the caudal region of the mandibular ramus. It meets the surangular dorsally below the mandibular articulation and probably sends the retroarticular process but this region is broken off and missing.

#### Postcranial elements

Preserved postcranial elements (Fig 4, S3 Fig, see S1 Table for measurements) are partially disarticulated and some are broken into fragments. Most of the axial skeletons are missing except for the last sacral vertebra and nine proximal caudal vertebrae with mostly disarticulated chevrons. The last sacral is articulated with the two proximalmost caudals but disarticulated from the other seven caudals. These seven caudals are not the third to ninth caudals, but considering their size and shape it is likely that they are proximal caudals. The pectoral girdle and forearms are almost entirely missing except for the right scapula and humerus both of which are only partially preserved. There is also a small fragment of bone which might be a part of coracoid. The pelvic girdle is relatively well preserved and although there are no missing elements, they are generally incomplete and some are only represented by small fragments. The preserved hind limb elements include both femora, left metatarsus with distal tarsals, partial digit I, III, and IV. The distal portions of the unguals are missing in the first and fourth digits whereas the third digit has only two proximal phalanges (III-1 and III-2) with the proximal half of phalanx III-3.

Only the last sacral vertebra is preserved and articulated with the first and second caudal vertebrae (Fig 4G). All of the preserved vertebrae retain unclosed neurocentral sutures. The centrum of the sacral vertebra has a large pleurocoel and shows caudal expansion increasing the height to meet the first caudal vertebra. There is a shallow craniocaudally elongate and groove-like depression on the ventral surface of the centrum. This depression covers the entire craniocaudal length of the centrum and is narrow at the cranial end and becomes wider caudally. On each side, there is one infraprezygapophyseal fossa and a slightly larger and triangular supraprezygapophyseal fossa, which respectively corresponds to the centroprezygapophyseal and spinoprezygapophyseal fossa of sauropods [70]. They are also separated from each other by a prominent lamina. In the case of sauropods, this lamina is known as prezygodiapophyseal lamina [71]. The robust sacral rib broadens before it meets the ilium. The length of the sacral rib is more than 1.5 times that of the centrum. The neural spine is almost completely missing but there is a pair of small fossae near the base.

The spool-shaped first caudal vertebra (Fig 4G) has a cranial articular end that is smaller than the caudal end. The centrum of the first caudal has a pleurocoel that is slightly more elongate but less circular. The prezygapophyses face medially and extend to a half the length of the last sacral. There are elliptical dorsal and ventral infraprezygapophyseal fossae, but the thin lamina between them is poorly preserved so that it is not possible to recognize any additional fossa on this lamina. Dorsal to the infrazygapophyseal fossae, there is a deep supraprezygapophyseal fossa which is triangle-shaped as the one on the last sacral but larger. The transverse process is long and oriented caudolaterally but also shows slight dorsal orientation. Ventral to the transverse process is a large and triangular infradiapophyseal fossa which is also known in *Nankangia* [15]. The supraprezygapophyseal fossa and infradiapophyseal fossa get smaller in the following vertebrae but are present on every preserved caudal vertebra. There is a small protuberance with two low and craniocaudally elongated ridges on the caudoventral surface of the centrum. The second caudal vertebra has a concave cranial articular surface and flat caudal articular surface but the latter is poorly preserved. The craniocaudal length of the centrum is slightly shorter than that of the first caudal. The prezygapophyses extend over the mid length of the preceding centrum, but the cranial tip of the left prezygapophysis is missing. The second caudal also has dorsal and ventral infraprezygapophyseal fossae, but the lamina between them is not preserved on the right side and it is barely visible on the left. It is not certain whether there is a middle infraprezygapophyseal fossa. The transverse process of the second caudal is sub-horizontal unlike that of the first caudal which is oriented caudolaterally. The two ridges on the ventral surface of the centrum are almost invisible. The third caudal vertebra is not preserved except for the prezygapophyses that reach the mid length of the centrum of the second caudal. The remaining seven caudal vertebrae (Fig 4C) that are disarticulated with the proximalmost three caudals are articulated with each other. For convenience, these seven caudals are each designated here as caudal A to caudal G in a proximal to distal sequence. Although we do not know their exact positions, they are likely to be proximal caudals judging by their sizes and shape although three distalmost caudals may represent transitional or mid-caudals because the pleurocoels become substantially smaller than those of preceding caudals. Most of the seven caudals are heavily eroded on the right side in contrast to the relatively well preserved left side. Caudal A has three infraprezygapophyseal fossae. The middle infraprezygapophyseal fossa is slit-like and much smaller than the dorsal and ventral infraprezygapophyseal fossae. The neural spine is similar in morphology to that of the first caudal although it is missing its dorsal tip as well. As in the second caudal, the two ridges on the ventral surface of the centrum are extremely low and this is also the case for caudal B. Caudals B to G have only one infraprezygapophyseal fossa except for caudal E which has two infraprezygapophyseal fossae on the right side. Caudal B, in particular, has broken remnants of this lamina as a small bump and a ridge. Caudal B is generally similar in morphology to caudal A but the shape of its pleurocoel is more elliptical. Caudals C and D have a somewhat smaller infraprezygapophyseal fossa and pleurocoel compared to caudal B. The ventral surface of caudal C is obliterated, but caudal D has the two ventral ridges that are more prominent than those of preceding caudals on its centrum. These ridges become more pronounced in the following caudals. In caudal E, the centrum becomes low but its craniocaudal length is nearly the same as that of caudal D. The cranial articular surface is concave and the caudal one is obscured by matrix. Each pleurocoel is greatly reduced in size compared to that of preceding caudals. Caudal F has a pair of pleurocoels which are especially minute and almost indistinguishable. The caudal half of caudal G is missing. On each side, the centrum bears an elliptical pleurocoel, which is larger than that of caudals E and F. The infraprezygapophyseal fossae of caudal G are asymmetrical in size, the right one being much larger.

Fragments of chevrons are preserved, but they are disarticulated from the caudal vertebrae except for a small fragment which is articulated with caudals C and D. However, it does not provide much information in terms of its morphology. The preserved chevrons are craniocaudally narrow and proximodistally elongate. The most complete chevron (Fig 4D) is also the largest. It has a proximal articular surface, which is concave but the distal part is missing.

The scapula (Fig 4E and 4F) is not fused with the coracoid and has a laterally everted acromion process whose dorsal surface is flat. The distal part of the scapular blade is missing, so the exact length of the scapula or whether there is a distal expansion is uncertain. The cross section of the preserved scapular blade is an inverted tear shaped due to the relatively thick dorsal region. The glenoid is nearly flat and directed ventrally. The humerus (S3C Fig) has a round head which is medially expanded. The deltopectoral crest is broken off and the distal humerus is entirely missing.

The ilium is dolichoiliac and has a straight dorsal margin (Fig 4B). The right ilium is fragmentary preserving only the acetabular area and ventral region of the postacetabular process (S3D Fig). The left ilium, however, is more complete but missing the caudal end of the postacetabular process. The preacetabular process has a round ventral margin and extends ventrally to the level below the dorsal margin of the acetabulum. A shallow cuppedicus fossa is present but not visible in lateral view. There is no supracetabular crest and an antitrochanter is weakly developed. The straight brevis shelf faces ventrally, which is not visible in lateral view. At its caudal end is the brevis fossa which is shallow but broad. The pubic peduncle is craniocaudally longer than the ichiadic peduncle unlike that of *Rinchenia mongoliensis* or *Heyuannia yanshini*. The pubis (Fig 4H) is greatly concave cranially and the articular surface for the ilium is slightly depressed. Unfortunately, the pubic boot is missing in both pubes. The cross section of the pubic shaft is sub-triangular. The pubic apron is thin and narrow. The caudally concave ischium (Fig 3I) is similar in morphology to other oviraptorids [4, 40, 72]. The medial surface of the ischium is flat in contrast to the lateral surface that has a concavity due to the obturator process. The thin obturator process is well developed but incomplete, so its exact shape cannot be inferred.

Both femora (Fig 4J and 4L, S3A and S3B Fig) are almost completely preserved. The femoral head is nearly perpendicular to the shaft and the femoral neck is indistinct. A shallow depression separates the femoral head from the large greater trochanter which is also detached from the finger-like cranial trochanter by a prominent furrow. The shaft of the femur is moderately concave caudally and there is no sign of a fourth trochanter. The two distal condyles are well separated from each other by the large popliteal fossa. The lateral condyle extends ventrally below the level of the medial condyle. The tibiofibular crest is well developed and extends caudally beyond the caudal margin of the medial condyle (Fig 4J). A weakly developed medial epicondyle is present on the craniomedial surface (Fig 4L, S3B Fig). The distal tarsals are not fused with the metatarsals but closely attached to them (Fig 4K). These tarsals are deeper at the plantar extremity and each has a flat proximal surface. The size of the two tarsals are comparable, but distal tarsal 3 is deeper than distal tarsal 4. Distal tarsal 3 covers metatarsals II and III, but distal tarsal 4 only covers metatarsal IV. The metatarsals (Fig 4K, 4M and 4N) do not show the arctometatarsalian condition and every metatarsal has a pair of ligament pits. Metatarsal I (S3E Fig) is strongly reduced and not articulated with the rest of the metatarsals. It has a dorsoplantar expansion at the middle. Its articular surface for the phalanx I-1 is triangular in distal view. The medial ligament pit is larger and deeper than the lateral one which is just a shallow depression. Metatarsal II is straight and slightly shorter than metatarsal IV. In proximal view, the articular surface of metatarsal II is the widest. Metatarsal II becomes proximally wider in dorsal view but the reverse is true in plantar view. It has a distinct ridge on the plantar surface of its shaft. The distal articular condyle for phalanx II-1 is larger than that of metatarsal IV, and the lateral ligament pit of metatarsal II is larger than the medial one unlike the rest of the metatarsals. Metatarsal III is the longest and visible along its entire length although its mediolateral width becomes narrower proximally in dorsal view. At the proximoplantar end of the shaft, metatarsal III has a prominent mound whose plantar surface is flat. The distal condyle of metatarsal III bears two ridges on its plantar surface. The medial ridge is more prominent than the lateral one. Metatarsal IV is straight proximally but the distal shaft is laterally deflected to a small degree. The shaft of metatarsal IV displays a rather continuous mediolateral width. It has a mound-like process on the proximoplantar end similar to that of metatarsal III but it is much smaller. The distal condyle is smaller than those of metatarsals II and III and subtriangular in distal view. Metatarsal V (Fig 4K and 4M) is thin and slightly curved dorsally at the distal end so it almost touches the metatarsal IV. It also meets metatarsal IV and distal tarsal 4. Digits I and IV are nearly completely preserved only without their distal ends of the unguals. The preserved pedal phalanges (Fig 4M and 4N, S3E and S3F Fig) have symmetrical and ginglymoid interphalangeal joints. Phalanx I-1 (S3E Fig) is asymmetrical having a distinct medial projection on the proximal end. It has a pair of shallow ligament pits that are similar in size and shape. The ungual of digit I has a minute tubercle right ventral to the proximal articular surface. Digit II is not preserved. The digit III preserves complete proximal two phalanges and the proximal part of phalanx III-3. The phalanges of digits III and IV have a pit on the dorsal surface right proximal to the distal end for the flexor muscles. A pair of similar-sized deep ligament pits are present on the medial and lateral surfaces of the distal condyles of phalanges III-1 and III-2. Pedal digit IV has five phalanges including an ungual (S3F Fig). They have asymmetrical ligament pits, medial ones being larger and deeper than the lateral ones. The distal tip of the ungual is missing. The ungual is slightly curved ventrally with two distinct grooves on the medial and lateral surfaces. A short dorsal lip at the proximal end extends over phalanx IV-4.

### Phylogenetic analysis

**Fig 5:**
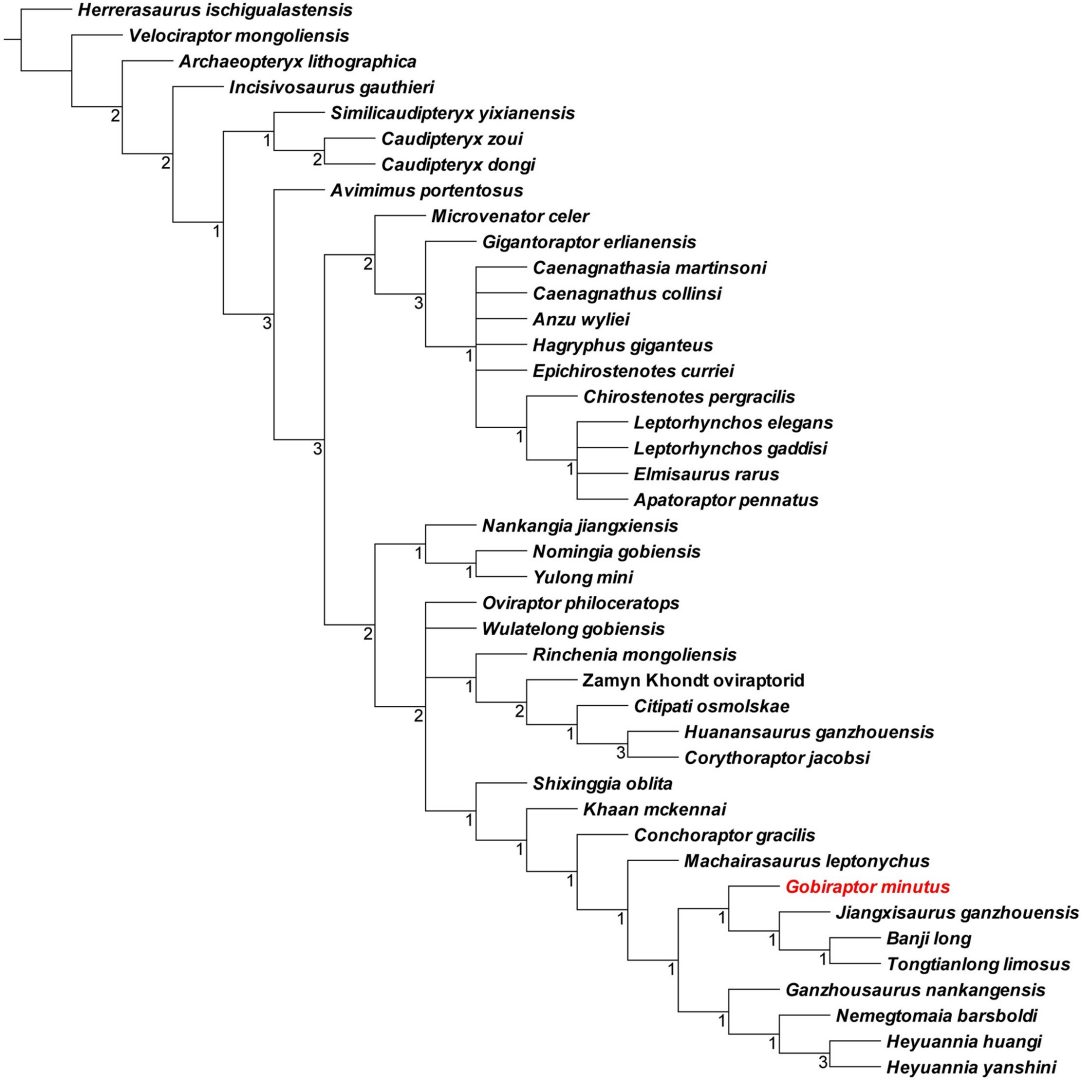
Strict consensus (CI: 0.448, RI: 0.647) of 24 most parsimonious trees of 652 steps obtained by TNT based on the data matrix of 42 taxa and 257 characters. Numbers at each node indicate Bremer support values.

The topology of the strict consensus tree (Fig 5) is generally similar to that of Lü et al. [20] with a better-resolved Caenagnathidae. The Mongolian oviraptorids are scattered across the subclades of Oviraptoridae, some of them being closer to those from geographically far regions than other Mongolian species. This is also the case for the oviraptorid taxa from the Nanxiong Formation of Ganzhou in southern China [18–20]. In addition, the strict consensus tree shows that *Gobiraptor minutus* belongs to the Oviraptoridae being the sister taxon to a clade composed of three Ganzhou oviraptorids: *Jiangxisaurus ganzhouensis* [17], *Banji long* [14], and *Tongtianlong limosus* [19]. These three Ganzhou taxa and *Gobiraptor* also form a small clade which is supported by the following three synapomorphies: premaxillae that have a significant ventral projection below the ventral margin of the maxillae (character 7, state 1), a vomer that is level with other palatal elements (character 222, state 0), and the same pattern of the distal ends of the shafts of metatarsals II and IV with a straight metatarsal II and a laterally deflected metatarsal IV (character 252, state 2).

### Osteohistology of *Gobiraptor minutus*

**Fig 6:**
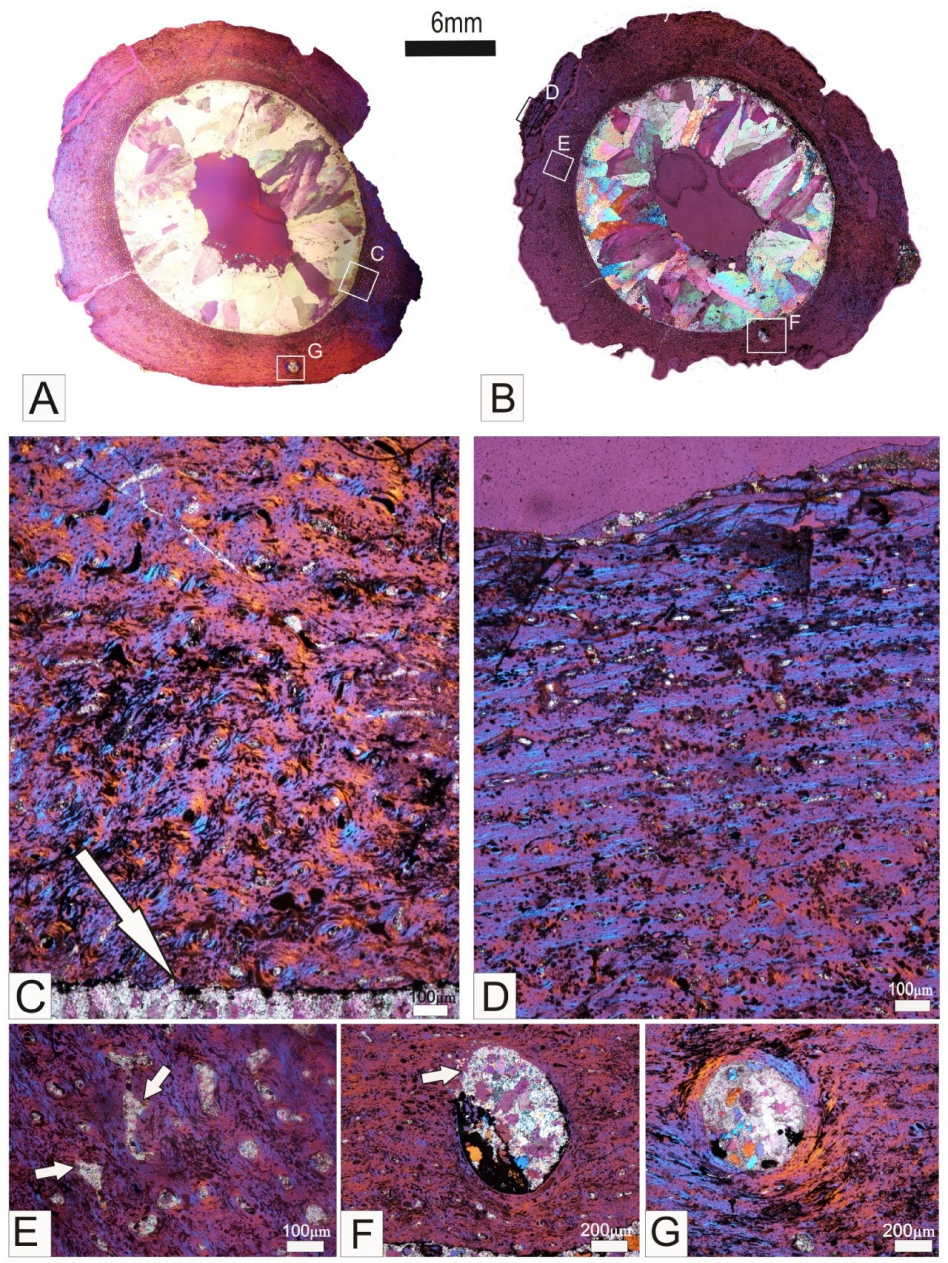
Osteohistology of the femur of the holotype specimen (MPC-D 102/111) of *Gobiraptor minutus* gen. et sp. nov. Transverse sections from the mid-shaft of the right femur. 25 microns (A) and 30 microns (B) thick thin sections. The maximum diameter of the cross section is ~24mm. A distinct layer of compact bone surrounds the large vacant medullary cavity. Framed regions indicate the location of higher magnification images. (C) A higher magnification of the framed region in A showing the fibrolamellar bone tissue, and detail of the endosteal region of the bone wall. Note the abundant primary osteons located in the woven bone matrix. The arrow indicates the resorptive endosteal margin of the bone wall. (D) A higher magnification image of the framed region in B showing the more lamellar organization of the bone matrix, and a laminar arrangement of the vascular canals. (E) A higher magnification of the framed region in B showing the secondarily enlarged canals. The arrows point to the uneven resorptive margins of the canals. (F-G) Large nutrient foramina in the bone wall. The arrow in F indicates the region of active bone resorption without a lining of lamellar bone.

Although there are some diagenetic alterations in the bone tissue, the histological structure is still reasonably well preserved in both the 25 micron (Fig 6A) and the 30 micron femoral thin sections (Fig 6B). The maximum cross sectional width of the bone is about ~24 mm. A narrow compact bone wall (~4 mm) surrounds a large vacant medullary cavity. The bone wall is comprised essentially of fibrolamellar bone tissue (Fig 6C). The woven bone matrix of the bone wall is inundated by many canals (that house vascular tissue, as well as other connective tissue) [73]. The canals tend to have variable orientations that range from longitudinal to more reticular arrangements (relative to the long axis of the bone). In localized areas the more recently formed periosteal bone (nearest the peripheral margin), appears to transition to a lamellar bone tissue (Fig 6D), which is indicative of a change to a slower rate of bone deposition. In a small section of the lateral bone wall the vascular canals follow a radial transect from the endosteal region towards the periosteal region (although the outermost part of the bone wall is not preserved). This arrangement most likely corresponds to a muscle attachment site [54]. Many well-formed primary osteons are present in the cortex (Fig 6C), although many of the canals of the primary osteons formed during earlier stages of ontogeny (nearest the medullary cavity) are secondarily enlarged due to resorption (Fig 6E). In the Fig 6A and 6B, a distinctive large nutrient foramen is evident in the compacta (see Fig 6F and 6G for magnified views). The foramen in Fig 6B is located closer to the endosteal margin of the bone wall, and the higher magnification image shows that it is partially lined by lamellar bone whilst in other parts the edges of the lumen are uneven and appears resorptive (Fig 6F). The foramen in the thinner section (Fig 6A) occurs more towards the periosteal region, and is completely lined by a narrow band of lamellar bone tissue (Fig 6G). The medullary cavity is large, and it has a distinctive uneven, resorptive margin (Fig 6A–6C and 6F), suggesting that medullary expansion was still underway. It is noteworthy that secondary reconstruction is at an early stage of development with many canals secondarily enlarged (Fig 6E), but there are no completely formed secondary osteons present in the compacta [54]. No growth marks (annuli or lines of arrested growth) are present in the compacta.

## Discussion

### Insights from the femoral osteohistology of *Gobiraptor minutus*

The overall primary nature of the bone compacta suggests that the holotype specimen (MPC-D 102/111) of *Gobiraptor minutus* was at an early stage of ontogeny. This can be inferred from the following osteohistological characters: abundant fibrolamellar bone tissue, the uneven osteogenic periosteal margin and the resorptive endosteal margin of the bone wall, and the early onset of secondary reconstruction without any fully formed secondary osteons (see Fig 6). This histological finding is congruent with the anatomical observations of closed neurocentral sutures of the preserved vertebrae.

### Ontogenetic stage and diagnostic characters of *Gobiraptor minutus*

Although the holotype specimen (MPC-D 102/111) of *Gobiraptor minutus* is probably a very young individual, the diagnostic characters of this taxon are unlikely to be related to its ontogeny. First, even though no complete ontogenetic variations in any oviraptorid taxon are known, some studies on very young individuals or embryos revealed that oviraptorids might be precocial animals having many craniomandibular characters also shown in adults [39, 74]. Secondly, according to the list of ontogenetic variations previously reported for oviraptorids [75], MPC-D 102/111 has a number of characters known in mature oviraptorids such as an inclined ventral margin of the maxilla, an angle of roughly 90 degrees between the jugal process of the maxilla and the descending process of the lacrimal, a spool-like sacral centrum, and the presence of a brevis fossa. All diagnostic characters for *Gobiraptor minutus* are not relevant to this list as well. For individual diagnostic characters, the flat articular surface on the quadrate for the quadratojugal is unique in this specimen, and it has been reported that an embryonic oviraptorid skeleton has a concavity on this surface [75]. A convex articular surface on the quadrate for the quadratojugal is known in *Nemegtomaia* [47]. However, given the very slight difference between lengths of dentaries (0.9:1 compared to MPC-D 107/15), it is highly doubtful that *Gobiraptor* would develop this character later in ontogeny. The relatively elongated rostral region of the dentary in *Gobiraptor* is also not likely to be an ontogenetic variation. This region in a presumably juvenile specimen of *Banji long* is very short [14], and it is fairly long in seemingly more mature oviraptorids [20, 49]. The thickened rostrodorsal end of the mandibular symphysis, along with small occlusal foramina on each lingual shelf defined by a shallow lingual ridge in *Gobiraptor* is not known in any other oviraptorid, both in young and mature specimens. Thus, above characters are best explained as autapomorphies rather than in an ontogenetic context. The symphyseal ventral process of *Banji* is prominent even though it is represented by a possibly juvenile specimen. This supports that the absence of the same process in *Gobiraptor* is probably not because of its young age. The coronoid bone in oviraptorids thus far has been reported only in the apparently mature holotype specimen of *Citipati* [67]. Its presence and positioning of the rostral end in *Gobiraptor* are, therefore, likely to be taxonomic variations. The cranial trochanter of the femur in *Gobiarptor* is completely ossified, which makes it improbable that it would be fused with the greater trochanter in later ontogenetic stages of this taxon.

### Implications of the unique mandibular morphology of *Gobiraptor minutus*

The mandible of *Gobiraptor* (Figs 2E–2K and 3, S2D–S2F Fig) has typical oviraptorid characters such as a short and deep dentary, a tall external mandibular fenestra, a prominent coronoid process, and a rostroventral process of surangular protruding into the external mandibular fenestra. However, the mandibular symphysis of *Gobiraptor* is very unusual in that its rostrodorsal end is extremely thickened by the dorsal surface which caudally expands. This peculiar morphology of the mandibular symphysis and the presence of occlusal foramina, lingual shelves and ridges are not known in other oviraptorids, but similar structures exist in derived caenagnathids [4, 26, 27, 33, 36, 51, 74]. This resemblance between *Gobiraptor* and derived caenagnathids may be a result of convergence. Nevertheless, the symphyseal region of *Gobiraptor* is certainly distinguished from that of caenagnathids namely by its strongly downturned form, the absence of proper occlusal grooves, unfused mandibular symphysis, the continuous surface of the symphyseal shelf, not as extensively developed lingual shelves, and much shallower lingual ridges. Thus, the mandibular structure of *Gobiraptor* may represent an intermediate state between that of other oviraptorids and derived caenagnathids as in case of *Gigantoraptor* which, however, does not have a thickened mandibular symphysis and lacks lingual ridges or occlusal foramina [51]. This distinct jaw morphology of *Gobiraptor* could be related to a specialized diet. While the diets of oviraptorosaurs are still puzzling, it has been suggested that oviraptorids were likely to be durophagous, eating eggs or mollusks [23, 41, 43, 44, 75] or they might be herbivores [15, 39, 76–78] and could be specialized for eating nuts and seeds like extant psittaciform birds [10] although Longrich et al. [77] argued that oviraptorid jaws were more suitable for shearing plants rather than crushing hard shells. Herbivory has been proposed for caenagnathids as well [26, 78], but Lamanna et al. [36] concluded that it is most appropriate to view caenagnathids as ecological generalists. Furthermore, Funston and Currie [27] noted that *Chirostenotes*, a derived caenagnathid, was probably an omnivore capable of processing meat as well as plant leaves with the sharp edges of rhamphothecae. Caenagnathids also possess hind limbs that are suited for a cursorial lifestyle [32, 79] that could have been helpful in chasing prey. On the contrary, the non-arctometatarsalian foot of *Gobiraptor* is not effective in fast running [80], meaning that active hunting is highly doubtful for this taxon. Instead, durophagy or granivory or possibly both modes of feeding would have been suitable for *Gobiraptor* judging by the unusual structure of the dentary. Hard food items could be crushed by its thickened mandibular symphysis with assistance from the lateral occlusal foramina on the lingual shelves and propalinal movements of the jaw joint. Consequently, *Gobiraptor* probably had a different diet and occupied a different specific dietary niche from derived caenagnathids or other Nemegt oviraptorids. The unique morphology of the mandible and the accordingly inferred specialized diet of *Gobiraptor* also indicate that different dietary strategies may be one of important factors linked with the remarkably high diversity of oviraptorids in the Nemegt Basin (*sensu* Eberth [62]). Future discoveries and works on more oviraptorid specimens will be of great help in estimating their exact feeding habits.

### Phylogenetic position of *Gobiraptor minutus*

The position of *Gobiraptor* on the strict consensus tree (Fig 5) indicates that *Gobiraptor* is a derived oviraptorid and closer to three Ganzhou oviraptorids *Jianxisaurus*, *Banji*, and *Tongtianlong* than to others from the Nemegt or Baruungoyot formations such as *Nemegtomaia* or *Conchoraptor*. This kind of discordance between the geographical and phylogenetic distances is prevalent among oviraptorids from Mongolia or southern China as shown by the strict consensus tree as well as in recent studies [8, 18–20], implying that sympatric speciation was not a major factor in the evolution of oviraptorids in these regions [10]. Although they form a distinct subclade, *Gobiraptor* is clearly distinguished from *Jianxisaurus*, *Banji*, and *Tongtianlong* which together form another subclade. Most notably, the morphology of the mandible, especially of the dentary, of *Gobiraptor* is distinctively different from those of the other three taxa. One of the most prominent differences is the extent to which the symphyseal region of the dentary is downturned. *Gobiraptor* has a greatly downturned symphyseal region but in case of *Jianxisaurus*, it is marginally downturned and it is nearly straight in *Banji* and *Tongtianlong* [14, 17, 19]. The femoral osteohistology of the holotype specimen (MPC-D 102/111) of *Gobiraptor minutus* (Fig 5) also suggests that it did not reach maturity before death. Therefore, it is highly unlikely that the morphological differences between *Gobiraptor* and its three closest relatives represent ontogenetic variation but are best explained by a rapid adaptive radiation of the Ganzhou oviraptorids [19]. However, the low Bremer support value of this subclade implies that it is weakly supported, and future studies may find alternative phylogenetic relationships among these four taxa. *Gobiraptor* also represents the first oviraptorid taxon from Altan Uul III. The absence of *Gobiraptor* specimens from other localities might be a result of sampling bias, but it has been noted that each species of Nemegt oviraptorids has occurred only in one locality in spite of high diversity [10, 77]. Thus, it appears to be reasonable to assume that most oviraptorid taxa, if not all, from the Nemegt Formation were separated from each other spatially or temporally as Funston et al. [10] indicated. The reason behind this is uncertain although niche partitioning [81–84] or high species turnover in a short time interval [85, 86] might have played a role [10]. The distant phylogenetic relationships among Nemegt oviraptorids, therefore, imply that the evolutionary history of this diverse family in the Nemegt region might be more complicated.

### Paleoecology and diversity of oviraptorids in the Nemegt Basin

The Nemegt and Baruungoyot formations in the Nemegt Basin are rich with oviraptorids [1, 2, 4, 42–44, 47–49] as well as other dinosaur taxa [50]. Whereas the Nemegt Formation was mostly formed by fluvial, alluvial plain, paludal, and lacustrine deposits indicating mesic environments [59, 60, 62], the Baruungoyot Formation includes eolian deposits in addition to those mentioned above and has been interpreted to represent drier environments [62, 87, 88]. Previous works showed that these two formations interfinger at Hermiin Tsav and Nemegt area forming successive stratifications [60, 62, 63, 89], the latter locality producing *Nemegtomaia* from the beds of both formations [49]. The distribution of oviraptorids in the Nemegt Basin is thus different from those of avimimids or Nemegt caenagnathids, which are known only from the Nemegt Formation [10, 11, 13, 33, 90, 91]. Although it was suggested that oviraptorids preferred xeric environments because of their abundance in the Baruungoyot and Djadochta formations [10, 33, 77], the presence of multiple oviraptorid taxa in the Nemegt Formation showed that they were also well adapted to wet environments [10]. The discovery of *Gobiraptor* and associated fragmentary oviraptorid specimens confirms this notion. In addition, oviraptorid diversity in the Nemegt Basin is increased by *Gobiraptor* to six taxa not including the unnamed Guriliin Tsav oviraptorid [10] although *Nomingia* is thought to be a possible caenagnathid [2, 4, 10] despite its phylogenetic position (Fig 5). The reason behind this remarkable diversity of oviraptorids is still a mystery, although it is apparent that they diversified in a short time span and prospered in both dry and wet environments.

## Conclusions

*Gobiraptor minutus* gen. et sp. nov. is a new derived oviraptorid represented by an incomplete skeleton including both cranial and postcranial elements. *Gobiraptor* is primarily distinguished from other oviraptorids by its dentary with the extremely thickened rostrodorsal end of the mandibular symphysis, lingual ridges and lingual shelves bearing occlusal foramina. The unique morphology of the mandible of *Gobiraptor* is probably closely related to a crushing-related feeding style and a specialized diet, which may have incorporated hard seeds or shelled organisms. Although *Gobiraptor* was recovered from the Nemegt Formation, its phylogenetic position showed a close relationship with three Ganzhou oviraptorids. The distant relationships among the Nemegt oviraptorids on the phylogenetic tree were reaffirmed in this study. Therefore, it is highly unlikely that the evolution of these unusually diverse animals was facilitated by a simple sympatric speciation. The presence of *Gobiraptor* in the Nemegt Formation, together with occurrences of other oviraptorids, also indicates that abundant oviraptorids lived in mesic environments and they were one of the most diverse and successful groups of dinosaurs in the Nemegt region.

## Supporting information

S1 Fig The site of Altan Uul III where the holotype specimen (MPC-D 102/111) of *Gobiraptor minutus* gen. et sp. nov. was found in 2008. (TIF)

S2 Fig Additional images of the cranial elements of the holotype specimen (MPC-D 102/111) of *Gobiraptor minutus* gen. et sp. nov. (A) Premaxillae and left maxilla in ventral view. (B) Right premaxilla in lateral view. (C) Right quadratojugal and quadrate in lateral view. (D) Rostral region of the mandible in caudal (D) view. (E-F) Caudal region of the right mandibular ramus in medial (E) and dorsal (F) views. Abbreviations: an, angular; ar, articular; cp, coronoid process; lgl, lateral mandibular glenoid; mgl, medial mandibular glenoid; mx, maxilla; pra, prearticular; qj, quadratojugal; sa, surangular; spl, splenial. Scale bar equals 1 cm.
(TIF)

S3 Fig Additional images of the postcranial elements of the holotype specimen (MPC-D 102/111) of *Gobiraptor minutus* gen. et sp. nov. (A-B) Left femur in caudal (A) and medial (B) views. (C) Right humerus in cranial view. (D) Right ilium in medial view. (E) Left metatarsal I and pedal digit I in medial view. (F) Left pedal digit IV in lateral view. Abbreviations: brs, brevis shelf; bvf, brevis fossa; fct, cranial trochanter of femur; fh, femoral head; hh, humeral head; isp, ischiadic peduncle; mep, medial epicondyle; mt I, metatarsal I; pf, popliteal fossa. Scale bar equals 5 cm.
(TIF)

S4 Fig Strict consensus of 24 most parsimonious trees of 652 steps with synapomorphies obtained by TNT. (TIF)

S1 Table Selected measurements of the holotype specimen (MPC-D 102/111) of *Gobiraptor minutus* gen. et sp. nov. (XLSX)

S1 Text Data matrix of Oviraptorosauria and outgroups used in this study (modified from Lü et al. [20]). (DOCX)
