# Supplementary figures and images for: A new baby oviraptorid dinosaur (Dinosauria: Theropoda) from the Upper Cretaceous Nemegt Formation of Mongolia
Source: PLoS One. 2019 Feb 6;14(2):e0210867. doi: 10.1371/journal.pone.0210867 (PMC6364893; doi:10.1371/journal.pone.0210867)

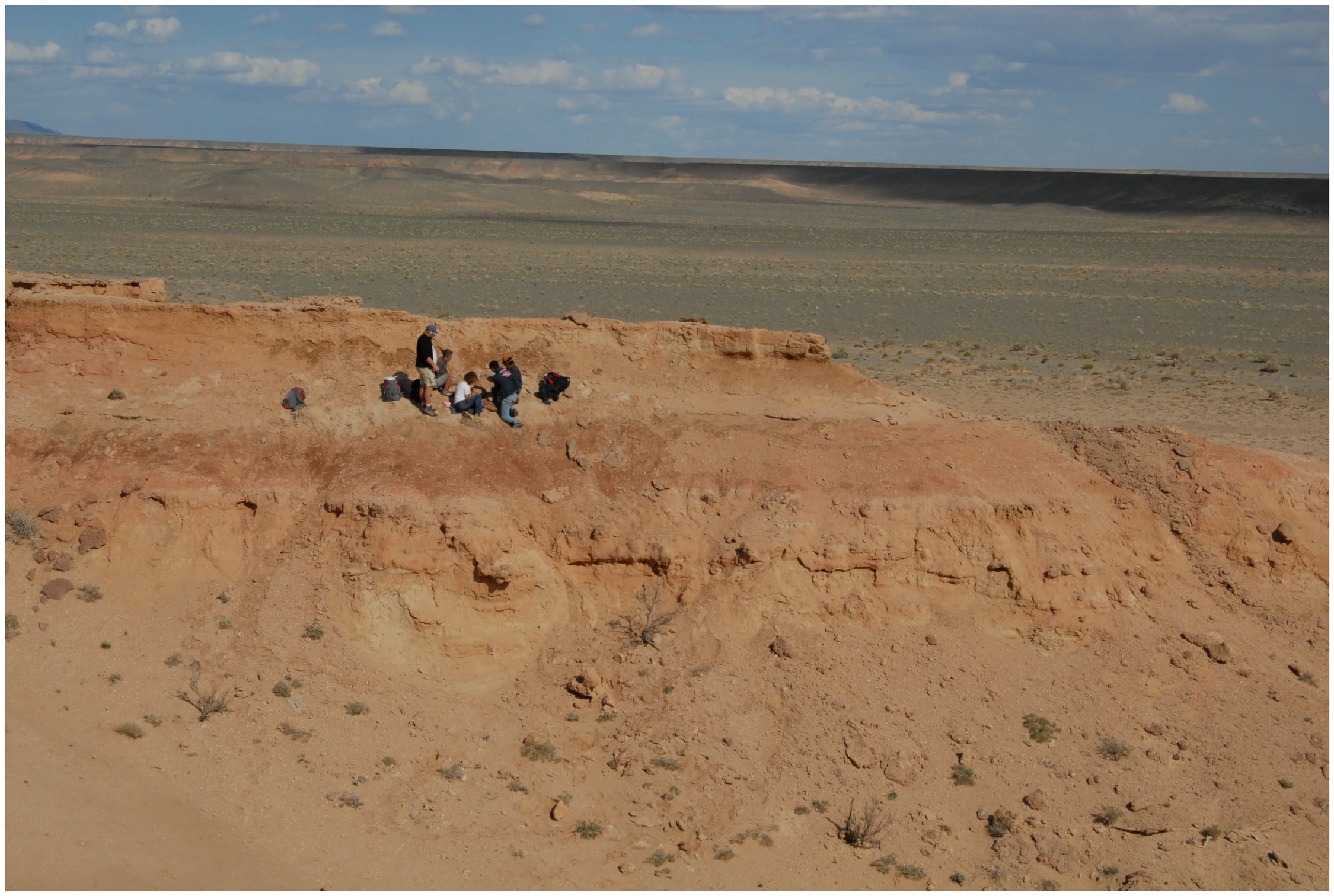

Supplement: S1 Fig — (TIF) [file pone.0210867.s001.tif]

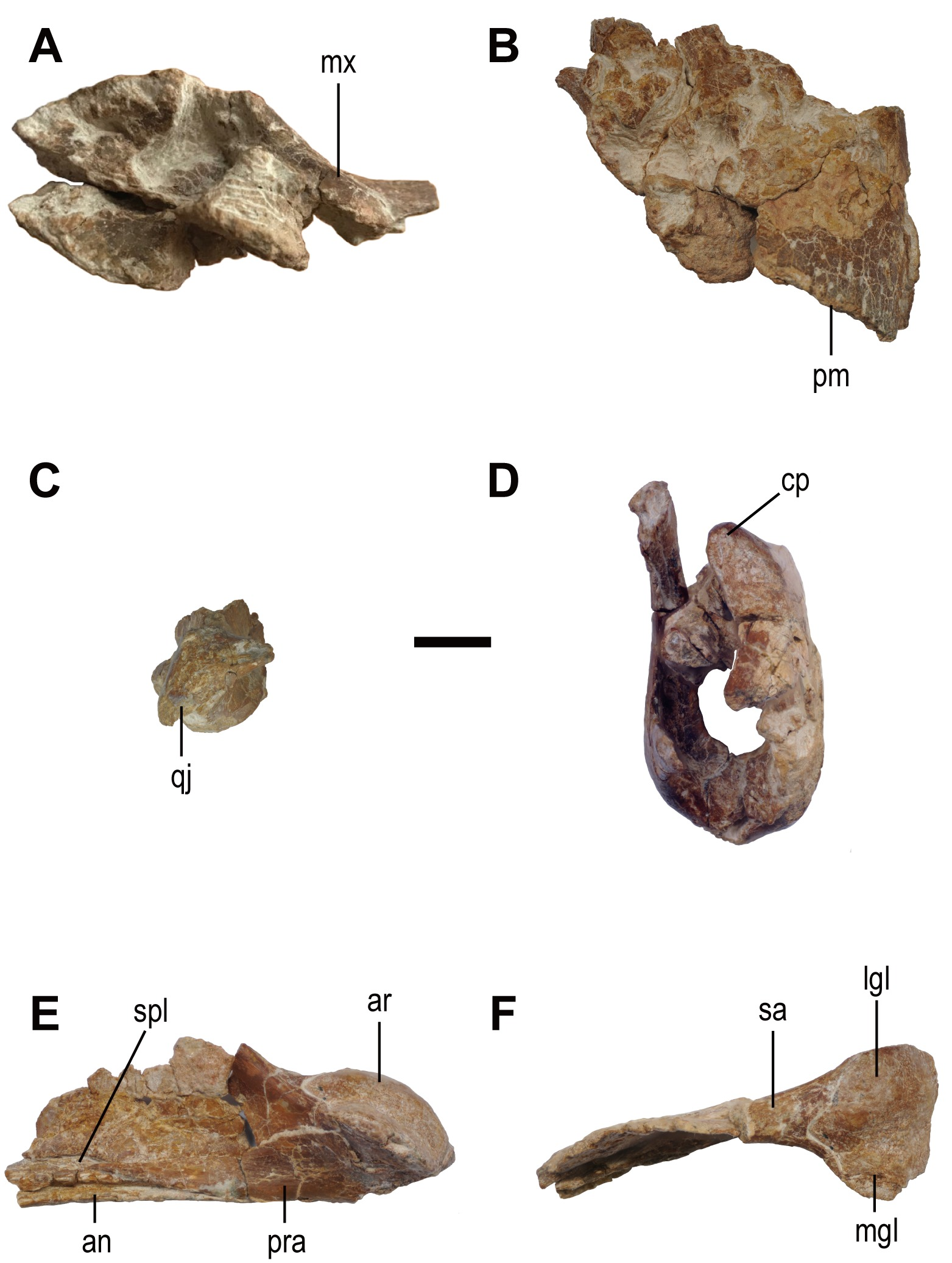

Supplement: S2 Fig — (A) Premaxillae and left maxilla in ventral view. (B) Right premaxilla in lateral view. (C) Right quadratojugal and quadrate in lateral view. (D) Rostral region of the mandible in caudal (D) view. (E-F) Caudal region of the right mandibular ramus in medial (E) and dorsal (F) views. Abbreviations: an, angular; ar, articular; cp, coronoid process; lgl, lateral mandibular glenoid; mgl, medial mandibular glenoid; mx, maxilla; pra, prearticular; qj, quadratojugal; sa, surangular; spl, splenial. Scale bar equals 1 cm. (TIF) [file pone.0210867.s002.tif]

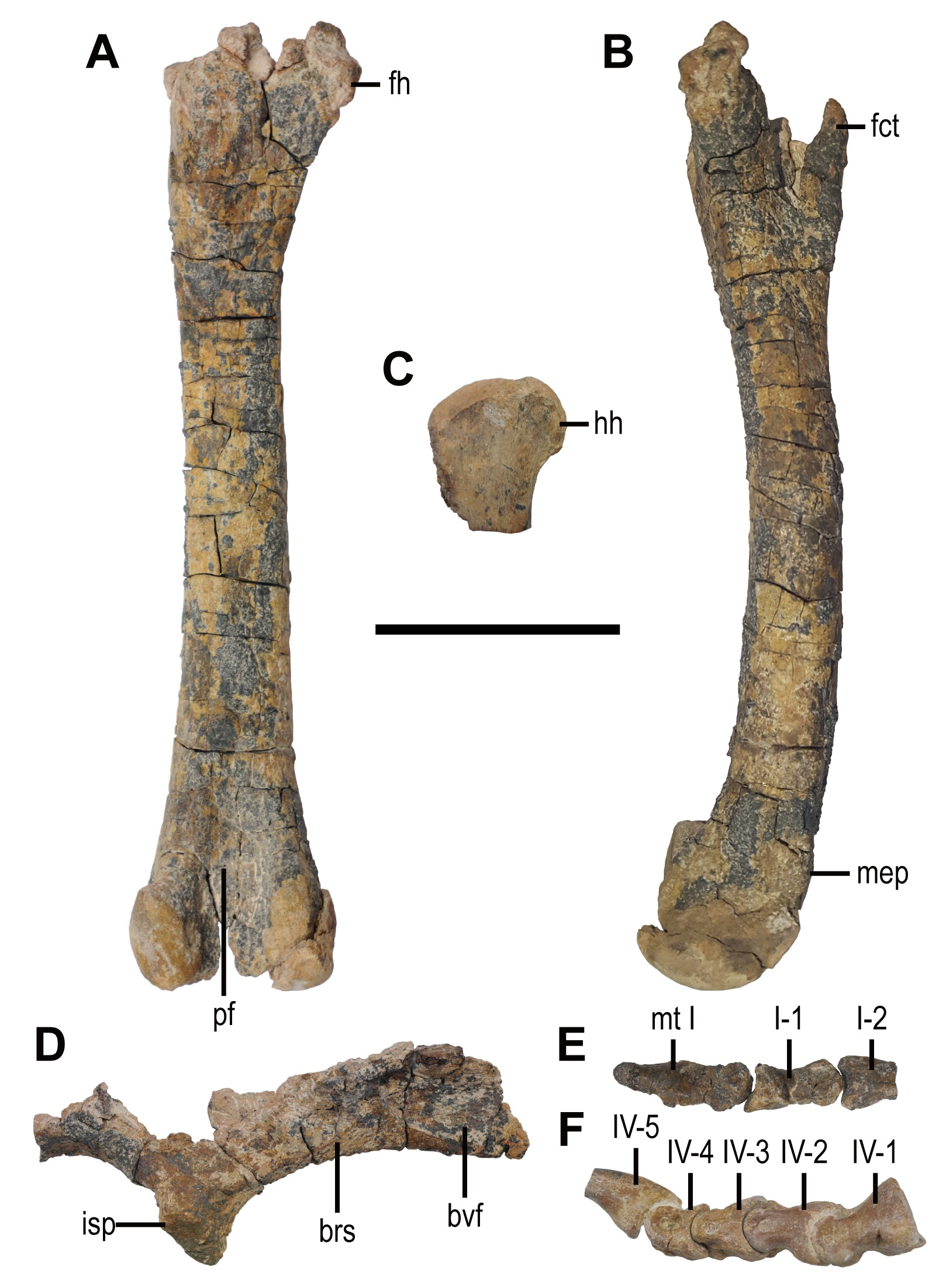

Supplement: S3 Fig — (A-B) Left femur in caudal (A) and medial (B) views. (C) Right humerus in cranial view. (D) Right ilium in medial view. (E) Left metatarsal I and pedal digit I in medial view. (F) Left pedal digit IV in lateral view. Abbreviations: brs, brevis shelf; bvf, brevis fossa; fct, cranial trochanter of femur; fh, femoral head; hh, humeral head; isp, ischiadic peduncle; mep, medial epicondyle; mt I, metatarsal I; pf, popliteal fossa. Scale bar equals 5 cm. (TIF) [file pone.0210867.s003.tif]

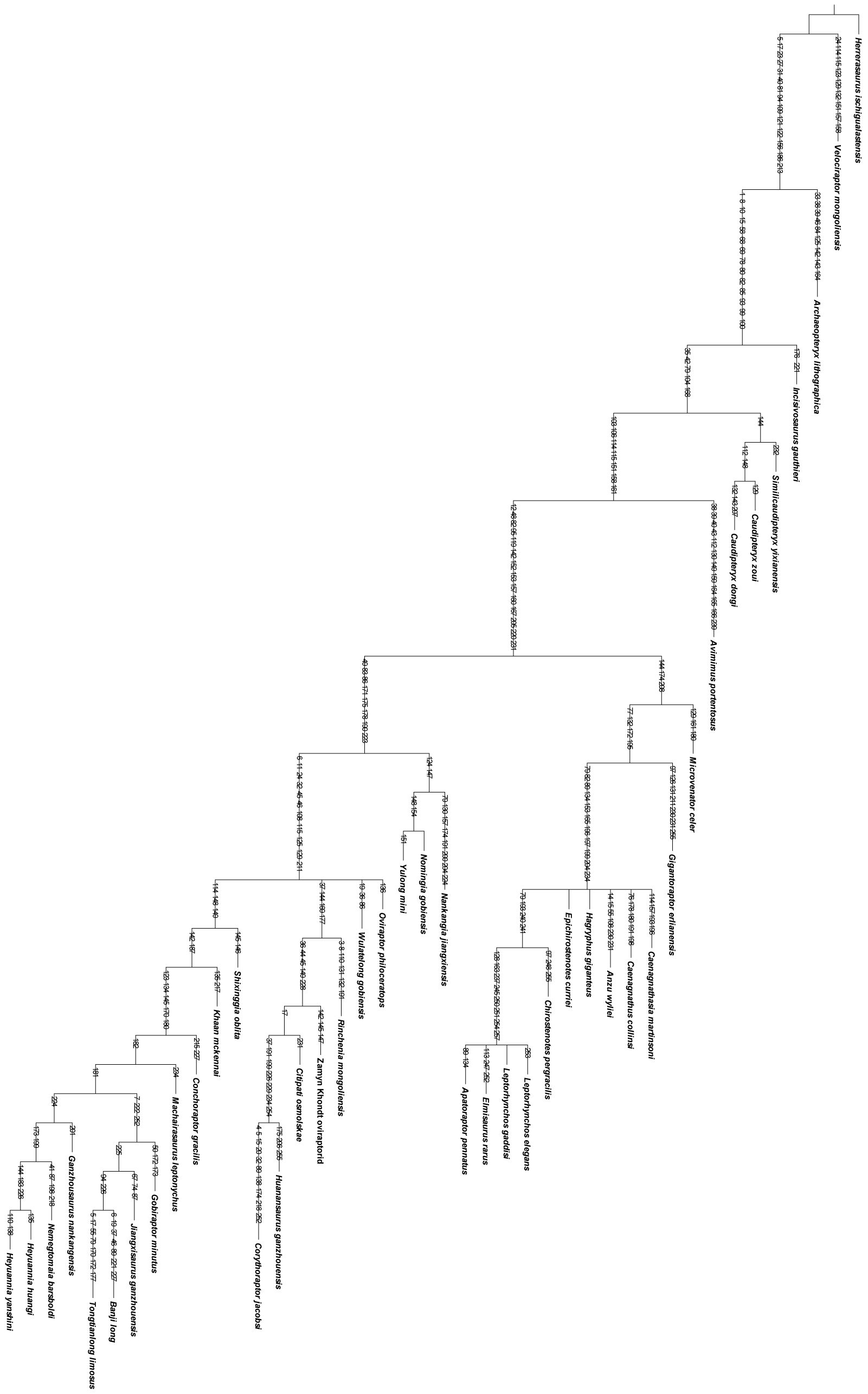

Supplement: S4 Fig — (TIF) [file pone.0210867.s004.tif]
