# Supplementary material for: A new baby oviraptorid dinosaur (Dinosauria: Theropoda) from the Upper Cretaceous Nemegt Formation of Mongolia
Source: PLoS One. 2019 Feb 6;14(2):e0210867. doi: 10.1371/journal.pone.0210867 (PMC6364893; doi:10.1371/journal.pone.0210867)
Supplement: S1 Text — (DOCX) [file pone.0210867.s006.docx]

**Data matrix of Oviraptorosauria and outgroups used in this study (modified from Lü et al. [1])**

Herrerasaurus_ischigualastensis
000100-0000000000000000000000000000000000000000000000000000000000000000000000000000010000000000000000000000010?0000000000100?0000101??000100000000-0000101000000000000100000?000?0000110010100??000??0000101??00??0000???00?1000?000000?00000???????000?0000?000?

Velociraptor_mongoliensis
000200-00000010100101001010001000000000010000000001000010000000000000000000000000000000001100000000000010010110111101101011111-1101011120211000011011112020011100010010000000000?0000000011000?0000??00100001000000000110011?000?00000000000000?????00000000?000?

Archaeopteryx_lithographica
000?10-000000101101001100010001?1000-1110??0?1??0020????000????0000001?1???1000010-10000011-01000000-10100??0101?00?110110010111001111111111011010-111020201?0100?1101200?0000?0?0?00000001000?0000??001?000000?010010?0?111??00?00000?0000?00??????00000000?000?

Incisivosaurus_gauthieri
100210-101000011?0111210011?01?00?00000?100010?0??200111?10?10?01??111?0101?2101110020??00011??11?1110011??????????????????????????????????????????????????????????????00000000100??????????00100000000000000???????1???????00???01000???????0???????????????000?

Caudipteryx_zoui
10?210-?01??0?1110?0?21?01?10???011000011100100???????????????????0????????0??1111??2???0001??????11??0220?????010????0?1??11???1011?1010200?0012110110?021100???1000121??0???0??0?00?001000?000?00000000?01000?0010???0101????0?00000?0000000?0??????000000?000?

Avimimus_portentosus
???????????0???????????????10110??1?-1121-101??00?101111?10???????????????0?1??111???01?????11000???-11?21000110?110??0???2?12100111?1?????00000?1110?10020101001111222100010000?0???????00??00?00????0??0000??0??10110??11110????????0?0?000010?????1000000?00??

Microvenator_celer
????????????????????????????????????????????????????????????????????????????1111?2?????11001????????????2???0??1?????????0????1?1011??1????0???1111?11?112???11101????????0101???001{01}?????00??0000000?0??????0?1??1???0??1?0???1?????0??00???????????????????????

Oviraptor_philoceratops
1???????????1?11?????21????????1??10000????0????????????????????????1?????????1?121022??10011???1??1???22???0??1????????????2???101101?212??????????????????????????????0???001001000000??0??1????????????????0??????????0??11?0?0???0??00???0???????????????000?

Rinchenia_mongoliensis
11102??0111110111???1211111111?11110100111000111111?11111-11?111111112111111111112102?01100111111111-11221110011?1010010102?22101000???????011011111101????111111??????1101?001011????????00111??????00000001????????1?10???1110?0?0?0???????????????????????000?

Citipati_osmolskae
1120211111111011011112111111111111111001110111?1??11111??101111?1111121?111111111210220110011111111110122???????1????????????????0?101?1???????????????????????????????11011001011?01010?0??1100000000000000100?????010100?01110?001000100?0?0??????00?0??00?000?

Zamyn_Khondt_oviraptorid
112021111111101111111211111111111111100111011111111111111-111111111112111111111112102201100111111111-11221110111?1010010102102101011011112101001210100111211111111000111101?001011?0000010??110?0000000000?0100???00?1?100??11???001?0??00??00???????????????000?

Khaan_mckennai
10201101?1111011?112121111111111?110000111000?111?111111???0??1?????1??????111111210210110011111111???1221110??1????00?0102??210??1101011210?0001100001?1211111?1100011100110010010010100111110?000?001000001000000?01?1100?1?10101000?0000{01}00??????00000000?000?

Conchoraptor_gracilis
10101101111110111112121111111111111000011100?1111111?1111-11111111111211111111111?102?01100111111111-11221110111?201001010112210111100111210100021000011121111111100011101?????????110???1??11????????????????0??????11?00?011?0?0000011?0??000?????00000000?000?

Machairasaurus_leptonychus
????????????????????????????????????????????????????????????????????????????????????????????????????????2???????????????????????????0011??????????????????????????????????????????111110?????????????????????00??????????????????????????1???????????????????????

Nemegtomaia_barsboldi
11100101111110111111121011111111?11000010101011?111111?1???11?11111112111111111112102?11100111111?11-11221110{02}11?201????????????????????????10002???00?1????11?????????10111101011?12?11??1111000000010000001000??0??1???1??11?1?1?0001?000??00??????????????000?

Heyuannia_huangi
??????????????????????????????????1???????????????????????????????????????????1??21021??1001????1???????21???21-12????1?????221?11000001?20???01?10100111?1???????????????1?1?1011112?2101?1?????????0?00000100?0?0?0101?00?1?11?0???0?00001????????000000000000?

Heyuannia_yanshini
????????1???????????????11?????1?1100001110?0111111???111???11?1??111?1???11111112102?01100110111111-1122?110011?201001010102210110000110000100121000011121111111100011?011?10101111212101111100000000000000100?0000?1?100001?1??0???0?000010?00000000000000?000?

Gigantoraptor_erlianensis
??????????????????????????????????????????????????????????????????????????11211112002011100111112?????1?2???????????0010?0??10?00000?11?1??1????????????1??1111111000?1???0001010000??????????0???1001001110100???0??????1?????110??112?10??????????010?000??011?

Caenagnathasia_martinsoni
????????????????????????????????????????????????????????????????????????????2101?1?????120??????????????2?1002?1?0?1???????????????????????????????????????10?1???????????0001???0????????????011?111?1??????????????????????????????0????????0??????????????????

Leptorhynchos_elegans
???????????????????????????????????????????????????????????????????????????12111?1?????1200???????1?????2?????????????????????????????????????????????????????????1111????0001???0???????0????0111121?10???????????1???????????0?????0??????1?????111100111111??1

Leptorhynchos_gaddisi
????????????????????????????????????????????????????????????????????????????21?1?{12}?????1{12}???????????????2?????????????????????????????????????????????????????????101??????0??????????????????0111121?1?????????????????????????????????????1????????????????????

Chirostenotes_pergracilis
??????????????????????????????????????????????????????????????????????????11211111002011200111112111-1??2????????111??????????10?????0????1??11??111101???111?1?1100111???00010100?100101000??01111211111111112?1111????????1??010???01??1000??111100001100100110

Caenagnathus_collinsi
??????????????????????????????????????????????????????????????????????????10210111002011200111111111-1??2?????????????1????????????????????????????????????111101?001?????00010101?1??????????110112101111111?????1????????01?0010??201??1????????????0???0??000?

Anzu_wyliei
?1??10-1100?1100??????????????????0?0000???????1011??100?10??1??0?011?????1121011?0020112001111111?1-11221110211?1?10010?02?12100010????1210???1?11?1?110211111111?????1?000010100000??01?0??0?10112111011111??1111?110101101?00101021201110??????1????0?????0000

Hagryphus_giganteus
????????????????????????????????????????????????????????????????????????????????????????????????????????????????????????????????????0010??????????????????????????????????????????000010??????????????????????2??????????????????????????1?????00??????????????1?

Elmisaurus_rarus
??????????????????????????????0?????????????????????????????????????????????????????????????????????????????0??10?????????2????1?????012???????????????????11?1???1111??0?????????000?10?0???????????????????11????1?????????????????????1??11111111111011120??11

Nomingia_gobiensis
????????????????????????????????????????????????????????????????????????????????????????????????????????????00?1?01?00101020????????????????1110110011111111111111??????????????????????1?00????????????????????111???0???1???1????????1??10?????????????????????

Epichirostenotes_curriei
??????????01?010??????1??????????????????????????????110?10?1??0?????????????????????????????????????????????2?1?111??1???2?????????????????????????????021?????????????????????????????1??????????????????????111??0?0???1???????????????10??0??????????????????

Banji_long
11?010-111???0111102121111111011??111001?????0??11?1111?????????1111121?0111??11?21021??000110??1?11??122?1????????????????????????????????????????????????????????????11111001011??????????110??????0?00000????????????????00?0?000?0???????0???????????????????

Caudipteryx_dongi
???????????????????????????????????????????????????????????????????????????????????????????????????????????????01?0?????????????0010?1?10200?011?11011000211?????100012????????????00?001000?????????????????01?10?????0??1?????????????00000???????000000000????

Ganzhousaurus_nankangensis
???????????????????????????????????????????????????????????????????????????1{01}111?210?{12}011001?????111????2????????????0???0????????????????????????????????????????0001????1?001?11???????1????????0?001?10?0???????0???????????111???0??????????????000000000????

Jiangxisaurus_ganzhouensis
1??????????1?????????????11???????1?0001????????112?????1-1?????1?01121??011111112102111100111111?????122??10??11??1??1?????221?1110?0120????0?????1???????????????????1??110010111?2?????????111??2?0100000100??????1?1?0?????0011?00??00???????????????????????

Nankangia_jiangxiensis
???????????????????????????????????????????????????????????????????????????11101?2??????100?????????????2??????1???1??1??0?0111001?????????01110110111111211001111????????1101??0???????1?00??11?????0?1???1????11???????01???11?0???0??0?00?????????????????????

Shixinggia_oblita
???????????????????????????????????????????????????????????????????????????????????????????????????????????????1?2?1??1?????????????????????110000-000?????1????1?????????????????????????01?????????????????????????????????????????????????????????????????????

Similicaudipteryx_yixianensis
????????????????????????????????????????????????????????????????????????????????????????????????????????????0?11?0?1?0??1?2???????1????????0?001?111110?????????0??0?12???????????????????00???????????????????????0???????????????????10?????0?????000?0000?????

Wulatelong_gobiensis
11?02101?11?1?1???02121?111?1??1?1110001???00??11????????????????1111????????????????101????1??11?????12???????1???100????2??????????1??????1100?1111111121???????000?11???????????0??????011????????????000????1????1?10?101????????????0??0??0????????00000????

Yulong_mini
102110-1110110110111121010111010?0100001110010?111110110???0????????0?????1111111210220110011{01}1111?1-1122?100??1????001?10??{12}??0000101?11110?110?100??0??1?1?0??11?001110?11001011?11000?100110??????0?0000010???????1??????1110?01000??00???0??????000?00000????

Huanansaurus_ganzhouensis
112021111111101101?11211111111111?110001110111?11100111?????1???11?11?????11111112102201100111111?????122?110211????????????????001101?112?????????????????????????????11011000011?01000????1?1?0????0100000110?????01???0??1??011??101?01????0?????????0000?1100

Tongtianlong_limosus
102021111111101?011212111111101111110001100001111?21110?????011?????1?????1111011210210110011011??????1221110201??01?01??0?02?1001?0???????01??????????1121??????1000?111010001001??????11??111??????01000001???10?101?010??1?100010001?0??0000??0??0??00??2?1??0

Corythoraptor_jacobsi
1121111111111001???21211111?1??01?11000???????????2?1???????????????1?????111111?2102???0001??????1???1221110211?111??1???2?????001101?11100110111110111121111111?1001111?11011011001010100011100000001000?0?00?1000?????110???0110110??01010?0000?100000002?10?0

Apatoraptor_pennatus
?????????????????????????????????????????????????????????????????????0?1???1211111002011100111111111-1??211002111?????????2?1111001001120211???1010???????????101?????????00010100010?10??????01?11?11111111110??????1?101?11??011??101?01????111????????????1001

Gobiraptor_minutus
??2??11?1?1???11??????????????????????????000??110??????????????1?11?21??1?11111?210??0?10011?11111?10122??????????1??1??????2?0?????????????0?0210?0?111?1001101?00011???10101011???????1????00001010100000???11010?1?????0?0?011??001?????0??????0?0000002100??

**Reference**

S1. Lü J, Li G, Kundrat M, Lee YN, Sun Z, Kobayashi Y, et al. High diversity of the Ganzhou Oviraptorid Fauna increased by a new "cassowary-like" crested species. Scientific Reports. 2017;7(1):6393. Epub 2017/07/29. doi: 10.1038/s41598-017-05016-6. PubMed PMID: 28751667; PubMed Central PMCID: PMCPMC5532250.
